# Supplementary material for: A compendium of genetic regulatory effects across pig tissues
Source: Nat Genet. 2024 Jan 4;56(1):112–23. doi: 10.1038/s41588-023-01585-7 (PMC10786720; doi:10.1038/s41588-023-01585-7)
Supplement: Supplementary file 1 — Supplementary Note and Supplementary Figs. 1–21. [file 41588_2023_1585_MOESM1_ESM.pdf]

nature genetics

Article https://doi.org/10.1038/s41588-023-01585-7

A compendium of genetic regulatory effects across pig tissues

In the format provided by the authors and unedited

The supplementary information contains:

Supplementary Note

Supplementary Figures

Supplementary References

Supplementary Tables 1-31 (in a separate Excel file)

7

Supplementary Note

Bioinformatics analysis of WGBS data

We downloaded 245 publicly available WGBS data (fastq files) from NCBI SRA and CNCB GSA by July 11th, 2021, representing 29 tissues, all generated using Illumina sequencers. We processed all these data using a uniform pipeline as described in the following. We first used FastQC (v0.11.9) (https://www.bioinformatics.babraham.ac.uk/projects/fastqc/) to evaluate the read quality and removed reads with low quality using Trim Galore (v0.4.5) (https://www.bioinformatics.babraham.ac.uk/projects/trim_galore/) with parameters: --max_n 15 --quality 20 --length 20 -e 0.1. We then aligned clean reads to the pig reference genome (Sscrofa11.1.100) using Bismark (v0.19.0) with default parameters and removed PCR duplicates from the mapped reads using the *deduplicate_bismark* function[1]. We obtained the methylation level of cytosines using the *bismark_methylation_extractor* (--ignore_r2 6) routine and removed CpG sites represented by less than five reads. Ultimately, we retained 182 samples with more than 10 million clean reads and unique mapping rate > 40%, representing 10 tissues and cell lines. We then removed data from corpus luteum as it had less than five samples. We visualized the variance in DNA methylation among samples using *t*-SNE, and removed samples from two cell lines, fibroblast and iPSC, as they were not clustered together or had no corresponding cell types in the current RNA-Seq dataset. Finally, we kept 166 samples from seven tissues to detect tissue-specific hypomethylated regions (HMR) and allele-specific methylation (ASM) loci. To detect tissue-specific HMR, we used SMART2 (v2.2.8)[2] with options: -t DeNovoDMR -MR 0.5 -AG 1.0 -MS 0.5 -ED 0.2 -SM 0.6 -CD 500 -CN 5 -SL 20 -PD 0.05 -PM 0.05 -AD 0.3. For ASM analysis, we employed Methpipe (v4.1.1)[3]. We first calculated the ASM score of each CpG site using the *allelicmeth* function and then obtained significant (FDR < 0.01) ASMs using the *amrfinder* function.

Tissue-specific gene expression and co-expression analysis

To explore the tissue specificity of gene expression, we only considered 34 tissues with over 40 RNA-Seq samples. We calculated eight distinct metrics to quantify the tissue specificity of gene expression, including Counts, Gini, JS, Roku, Shannon, Simpson, Spm and TAU (τ)[4]. To identify genes with specifically high expression in a tissue, we conducted differential gene expression analysis by comparing the target tissue with the other tissues based on TPM values using the limma (v3.51.2) R package[5]. We considered genes with LogFC > 2 and FDR < 0.05 as differentially expressed between tissues. To validate the tissue-specific genes, we downloaded 15 chromatin states predicted in 14 major pig tissues[6]. We conducted the chromatin state enrichment analysis of tissue-specific genes as described in ChromHMM (v1.22)[7]: (C/A)/(B/D), where A is the number of bases in the chromatin state, B is the number of bases in tissue-specific genes, C is the number of bases in both the chromatin state and tissue-specific genes, and D is the number of bases in the entire genome. We calculated the statistical significance of enrichment using Fisher's exact test. We performed gene co-expression analysis within each of the 34 tissues using five complementary methods, including WGCNA (v1.69)[8], ICA (v1.0.2)[9], PEER (v1.3)[10], MEGENA (v1.3.7)[11], and CEMiTool (v1.8.3)[12] with default parameters. We adjusted gene expression for hidden confounding factors using inferred PEER factors and genotype PCs (see below in molQTL mapping section) and then used the adjusted expression levels (Supplementary Fig. 8b-c) to infer gene co-expression modules by five methods separately[13]. We defined whether a gene is annotated in the GO database or not using the *getBM* function in the biomaRt R package (v2.48.0)[14]. We employed clusterProfiler (v4.0) to conduct the gene functional enrichment analysis based on the GO database[15]. We visualized the gene co-expression network using Gephi (v0.9.2)[16].

Bioinformatics analysis of Hi-C data

We downloaded Hi-C data of five samples from five different pig tissues (i.e., adipocyte, ear, embryo, liver and muscle) from NCBI SRA (by June 16th, 2021) to identify TAD and Hi-C contacts (Supplementary Table 9). After trimming adapter sequences and low-quality reads using Trim Galore (v0.6.7) (https://www.bioinformatics.babraham.ac.uk/projects/trim_galore/) with parameters: --q 20 --paired --max_n 15 --clip_R2 3, we aligned clean reads to the pig reference genome (Sscrofa11.1.100) using BWA (v0.7.17) with default settings[17]. We built Hi-C contact matrices at 500 kb resolution with Juicer (v1.6)[18] and identified TAD with Arrowhead (v1.22.01)[18]. We only considered TADs with FDR < 0.01 for the downstream analysis. We applied pyGenomeTracks (v3.6)[19] to visualize gene examples after converting .hic files to .cool files and then to .h5 files using hicConvertFormat (v3.7.1)[20].

Single-cell RNA-Seq and cell-type deconvolution analysis

We obtained 13 raw single-cell RNA-Seq data of five brain and eight lung samples from the CNGB Sequence Archive (CNSA) of the China National GeneBank DataBase (CNGBdb) under accessions: CNP0000686 and CNP0001486, respectively (by December 1st, 2021). We also obtained the processed single-cell RNA-Seq data of seven PBMC samples from USDA Ag Data Commons[21].

For five brain regions (frontal lobe: FL, parietal lobe: PL, temporal lobe: TL, occipital lobe: OL, and hypothalamus: HT), we used the Cell Ranger Single Cell Software Suite (v3.0.2) (https://support.10xgenomics.com/single-cell-gene-expression/software/pipelines/latest/what-is-cell-ranger) to process and analyze the raw 10x Genomics sequencing data. We used the *cellranger mkgtf* tool to keep all gene types in the gene annotation .gtf file (Ensembl v100) and the *cellranger mkref* tool to build a pig custom reference using both the generated GTF and Sscrofa11.1 (v100) genome assembly. We generated the raw count matrices using the *cellranger count* tool and then performed all subsequent analyses using the Seurat (v3.2.3) R package[22]. Overall, we obtained 2,465, 1,312, 4,974, 3,756, and 1,462 cells in FL, HT, OL, PL and TL, respectively, with the following criteria: 1) cells with gene counts over 200; and 2) cells with mitochondrial counts less than 5% of the total counts. We calculated gene expression using the *LogNormalize* method, implemented in the *Normalization* function and selected 2,000 genes as highly variable genes (HVG) using the *FindVariableFeatures* function with default parameters. Using the *FindIntegrationAnchors* and *IntegrateData* functions (dim = 1:20), we then integrated the five gene count matrices of HVG into one *Seurat* object, while correcting for batch effects. We applied the *ScaleData* function to scale the integrated data and then used it for principal components analysis (PCA) using the *RunPCA* function. Based on the elbow plot generated by the *JackStraw* function, we used the first 20 PCs for cell clustering and Uniform Manifold Approximation and Projection (UMAP) analysis with the *RunUMAP* function. We constructed a shared nearest neighbor (SNN) graph of cells with the *FindNeighbors* function and determined cell clusters with *FindClusters* at a resolution of 0.4. We then applied the UMAP algorithm to visualize the cell clustering. We utilized Azimuth (v0.4.0)[23] with a human motor cortex reference to label the cell clusters and assigned all cells to eight known brain cell types.

For lung tissues, we employed the DNBelab C Series scRNA analysis software to process the raw sequencing data generated from the DNBelab C4 sequencing platform[24], including creating a reference genome database and producing the gene expression matrix. We then applied the same pipeline and standards as described above to perform the following single-cell analysis. Collectively, 1,744, 966, 240, 1,317, 1,749, 10,000, 10,000, and 1,596 cells passed the quality control across eight samples. We assigned 35 cell types according to the human lung reference v1[25], implemented in Azimuth (v0.4.0)[23]. For PBMC, we directly used cell clusters provided by Herrera-Uribe et al.[21] for subsequent analysis.

Cell type deconvolution analysis

We selected four, five, and ten major cell types from the brain, lung, and PBMC, respectively, and then applied CIBERSORTx[26] to estimate the fraction of these cell types in bulk RNA-Seq samples from seven tissues, including brain, frontal cortex, hypothalamus, lung, blood, liver, and spleen. We extracted 150 cells from each cell cluster using the *subset* function, implemented in Seurat (v3.0.2)[22], to create a signature matrix using the CIBERSORTx online tool by the *custom* option with default parameters. We then uploaded the gene expression (TPM) matrix of bulk RNA-Seq samples as the mixture file. We imputed cell fractions based on the signature and mixture files by running the *Impute Cell Fractions* analysis with the *custom* mode and used the permutation test (100 times) to determine the significance level.

Genotype imputation reference panel

We downloaded 1,307 public WGS samples from NCBI SRA by March 18th, 2021, and newly generated an additional 510 WGS samples (Supplementary Table 2), representing five main pig populations worldwide, i.e., Suidae but not Sus scrofa (SUI, n=45), European wild boar (EUW, n=54), European domestic pig (EUD, n=855), Asian wild boar (ASW, n=80), and Asian domestic pig (ASD, n=783). We processed and analyzed the WGS data using a uniform pipeline, as described in the following. Briefly, we filtered the raw sequence reads by Trimmomatic (v0.39)[27], and then mapped clean reads to Sscrofa11.1 using BWA-MEM (v0.7.5a-r405) with default parameters[17]. We marked duplicated reads by Picard (v2.21.2) (http://broadinstitute.github.io/picard/). We removed 213 samples with low read depth (< 5x), one sample with low genome coverage (< 75%), and one sample with incomplete data. Finally, we kept 1,602 samples for jointly calling variants using the Genome Analysis Toolkit (GATK) (v4.1.4.1)[28] with parameters: QD> 2, MQ < 40, FS > 60, SOR > 3, MQRankSum < -12.5 and ReadPosRankSum < -8, yielding ~214 million SNPs. We removed SNPs with MAF < 0.01 and/or missing rate > 0.9 using bcftools (v1.9)[29] and employed Beagle (v5.1) to phase the filtered variants and impute sporadically missing genotypes[30]. Finally, a total of 42,523,218 SNPs were retained in the current version of PGRP (v1).

Genotyping and imputation of RNA-Seq samples

We employed the GATK (v4.0.8.1) to call SNPs at known loci in the dbSNP database (build 150), from 7,095 RNA-Seq samples, according to the recommended settings of the best practice guidelines[28]. We filtered out low-quality SNPs using the filtering option: FS > 30.0 & QD < 2.0 & DP < 4.0. We then imputed the filtered SNPs on autosomes to sequence level with Beagle (v5.1)[30] using haplotypes from the PGRP as a reference. Finally, we obtained 7,008 samples that were genotyped and imputed successfully. We filtered out variants with MAF < 0.05 and model-based imputation accuracy (DR^2) < 0.85, resulting in 3,087,268 SNPs for the molQTL mapping. We obtained 12,207 linkage disequilibrium (LD) independent SNPs using PLINK (v1.90)[31] with parameters: --indep pairwise 1000 5 0.2 [31], and then conducted a principal components (PC) analysis for all 7,008 samples using these LD-independent SNPs.

To evaluate the accuracy of genotype imputation from RNA-Seq, we collected a total of 725 additional samples with both genotypes (from WGS or 50K SNP array) and RNA-Seq data in seven populations/breeds that were independent of PGRP (Supplementary Table 3). We called SNPs from these RNA-Seq samples and imputed them to the sequence level using PGRP and then evaluated the imputation accuracy by comparing imputed genotypes from RNA-Seq to those 1) directly called from WGS data (i.e., RNA - WGS); and 2) imputed from the 50K SNP array (i.e., RNA - imputed SNP array). We measured the imputation accuracy using both concordance rate (CR) and genotype correlation (r^2)[32].

Breed prediction for RNA-Seq samples

There were 3,684 public RNA-Seq samples without breed information. We thus predicted their breed composition using the imputed SNPs *via* the pipeline summarized in Extended Data Fig. 1i. In brief, we estimated the breed composition of all 7,008 RNA-Seq samples based on 5,000 randomly selected LD-independent SNPs using ADMIXTURE (v1.3.0)[33]. According to the estimated ancestry proportion of samples with known breed information from three pure breeds (Duroc, Landrace, and Yorkshire) and two crossbred populations (Landrace×Yorkshire and Duroc×Landrace×Yorkshire) at different K values in the admixture analysis, we decided to choose K = 5 for further analysis. We identified samples with more than 10% ancestry proportion from ⩾ 2 ancestries as crossbreeds, while the remaining samples were considered as pure breeds. For crossbreeds and other pure breeds, we trained a machine learning model (implemented in RandomForest (v4.6-14) R package[34]) using samples with known breed labels to predict their breed information. We repeated the three-fold cross-validation 10 times to evaluate the prediction accuracy.

Detection of duplicated RNA-Seq samples within each tissue

To remove samples from the same individuals within each tissue, we first calculated the identity-by-state (IBS) distance among samples from LD-independent SNPs using PLINK (v1.90). IBS = (IBS2 + 0.5*IBS1) / (IBS0 + IBS1 + IBS2), where IBS0 is the number of non-missing variants with IBS = 0 (two different alleles), IBS1 is the number of non-missing variants with IBS = 1 (one shared allele), and IBS2 is the number of non-missing variants with IBS = 2 (two shared alleles). We set an IBS distance cutoff of 0.9 to deem samples as duplicates and kept the one with the largest number of expressed genes for subsequent analysis. We then removed an average of 54 duplicated samples (from 1 in the kidney to 455 in the blood) within each tissue, resulting in 5,457 samples in 34 tissues. To validate whether duplicate samples could be identified correctly, we calculated the IBS distance of 25 RNA-Seq samples from 9 individuals. We observed that samples from the same individual could be grouped together well according to the IBS cutoff of 0.9. In addition, we calculated Pearson's correlation of IBS calculated from imputed genotypes and those from WGS or SNP array from the same individuals.

Separate *cis*-QTL analysis for PCG and lncRNA

We separately conducted the molQTL mapping for PCG and lncRNA due to their differences in roles in regulating complex traits[35], *cis*-heritability and sequence conservations (Supplementary Fig. 21a). In addition, we also conducted the molQTL mapping using jointly normalized PCG and lncRNA expression data and compared the results with the ones that were obtained from separately normalized expression data. We found that the replication rate (π_1) of molQTL was close to 100% and the correlations of summary statistics (effect and *P* values) were higher than 0.95 across 34 tissues (Supplementary Fig. 21b-c). To further investigate whether adding known batch factors as covariates influences the molQTL mapping, we took muscle that has the largest sample size (n=1,321) as an example below. We performed the *cis*-eQTL mapping using the same linear regression model, implemented in TensorQTL, but considering the top ten PEER factors, top 10 genotype PCs and known confounders (e.g., age, sex and breed) as covariates. We considered sex as a categorical variable and the missing items as "unknown" class, while age as a continuous variable and the missing values were treated as the mean value of known age. Comparing these results to above ones where only PEER factors and genotype PCs were considered in the eQTL mapping, we found that the replication rates of *cis*-eQTL between them were 99.8%, while the Pearson's correlations of summary statistics were 0.96 for effect size and 0.98 for *P*-value.

cis-heritability estimation of gene expression

To understand the overall contribution of *cis*-genetic variants to variation in gene expression, we only considered SNPs within 1 Mb up- and down-stream of TSS. We then employed a linear mixed model to estimate the *cis*-heritability (*cis*-h^2) of each gene, while accounting for all the estimated covariates (i.e., genotype PCs and PEER factors). The linear mixed model is:

$$y = \mu + \sum_k \delta_k \cdot cov + g + e,$$

where y is the vector of normalized gene expression level, μ is the overall mean; δ_k is the fixed effect of the kth covariate (i.e., genotype PCs and PEER factors); g is the polygenic effect $\sim N(0, \mathbf{G}\sigma_g^2)$ where **G** is the local genotype relationship matrix (GRM) built by variants around ±1Mb of TSS of the target gene; and e is the residual. We estimated the genetic parameters using the restricted maximum likelihood (REML) method implemented in GCTA (v1.93.0)[36], and defined *cis*-h^2 with *P*-value < 0.05 (from the likelihood ratio) as significant. To further study the pairwise tissue similarity on gene regulation, we calculated the pairwise Pearson's correlation of *cis*-h^2 between tissues and then clustered tissues using the *hclust* function in R (v4.0.2).

Estimation of effect sizes of *cis*-eQTL

To quantitatively interpret the cellular regulatory events from the population data[37], we calculated the allelic fold change (aFC) of a *cis*-eQTL to quantify its effect size on gene expression using aFC (v0.3)[37], while taking account of the same covariates as done in the *cis*-eQTL mapping. We obtained the 95% confidence interval of aFC using the bootstrap method with the argument: --boot 100, and only kept *cis*-eQTL with a 95% confidence interval of aFC not overlapping with zero for further analysis.

Validation of *cis*-eQTL

To evaluate whether the complex genetic relatedness among samples affected *cis*-eQTL mapping using TensorQTL, we also analyzed each tissue's data with a mixed linear model, implemented in fastGWA (v1.93.0)[38], with a sparse genetic relationship matrix (GRM) that was constructed based on imputed genotypes for each tissue's samples. In addition to the GRM, we also included the genotype PCs and top ten PEER factors as above to account for the technical confounders. As done in the TensorQTL analysis, we only considered SNPs within the *cis*-window of a gene for *cis*-eQTL mapping based on fastGWA. We calculated the Pearson's correlation of the summary statistics from fastGWA and those from the TensorQTL, including Z-score (slope/slope_se), slope and significance levels (i.e., $-\log_{10}(P)$).

We conducted an internal validation of *cis*-eQTL for tissues that had a sample size ⩾ 80. For each tissue, we randomly and evenly divided the samples into two groups, and then conducted *cis*-eQTL mapping within each group separately, using TensorQTL. To measure the validation rate of *cis*-eQTL between groups, we calculated the π_1 statistic, defined as the proportion of *cis*-eQTL in group *i* that are significant in group *j* [39], using the *qvalue* method[40], and as well as the Pearson's correlation of estimated effect sizes (i.e., absolute z-scores from TensorQTL) of the *cis*-eQTL between groups.

To further validate the identified *cis*-eQTL, we examined two external datasets: 1) 179 animals with both 50K SNP array and blood RNA-Seq data at two-time points in a composite population of Landrace×(Duroc×Yorkshire); 2) 100 Duroc pigs with both WGS and RNA-Seq data from muscle, liver, and duodenum[41].

For the first validation dataset, we analyzed all the RNA-Seq data and imputed RNA-Seq genotypes to whole-genome sequence level based on the PGRP, using the same pipeline as described above. We also imputed genotypes from the 50K SNP array to sequence level. We then conducted the *cis*-eQTL mapping at each of the two-time points separately based on genotypes imputed from RNA-Seq and those imputed from 50K SNP array using the same pipeline as above, while considering the first five genotype PCs and ten PEER factors as covariates. To quantify the validation rate between the validation and discovery datasets, we calculated the π_1 statistic for the top *cis*-eQTL of genes identified in the validation dataset with those identified for blood in the PigGTEx discovery population. To further quantify the correlation of effect sizes of *cis*-eQTL between the discovery and validation datasets, we performed a meta-analysis of *cis*-eQTL using a multivariate adaptive shrinkage (MashR) method[42]. Following the same pipeline as described for the human GTEx[43], we used z-scores (i.e., slope/slope_se from TensorQTL) of the top *cis*-eQTL of genes as input and fitted the *mash* model using 1,000,000 random SNP-gene pairs being tested as null input. We obtained the estimated effect size (i.e., the posterior mean) of the *cis*-eQTL from the *mash* function and computed Spearman's correlation of those estimates between the discovery and validation datasets.

For the second validation dataset, we called genotypes directly from the WGS data but only considered 1,678,951 SNPs that overlapped those of the PigGTEx discovery dataset for *cis*-eQTL mapping. Using the same approach as described above, we performed *cis*-eQTL mapping for each of these three tissues using TensorQTL, while considering the first five genotype PCs and ten PEER factors as covariates. We then computed the π_1 statistic to quantify the validation rates of *cis*-eQTL between the discovery and validation datasets.

ASE is a complementary approach for *cis*-eQTL mapping at the individual level that is not affected by confounders among samples[44,45]. We utilized phASER (v1.1.1) to perform the haplotype-based ASE analysis[46]. First, we phased the variants from the bam and VCF files using phASER with parameters: --paired_end 1 --mapq 255 --baseq 10. We computed the mappability of each locus in the reference genome using GenMap (v1.3.0)[47] with 75 bp k-mers, allowing two mismatches (-K 75 -E 2). We then calculated the expression of the haplotype using phASER Gene AE (v1.2.0) with default settings and generated a haplotype expression matrix across all samples using the *phaser_expr_matrix.py* script. We then used the ASE results to validate *cis*-eQTL that had ⩾ 10 individuals with ASE data and ⩾ 8 reads for a given gene. We first calculated the ASE-level effect size (ASE aFC) using the script *phaser_cis_var.py* with default parameters and applied 10,000 bootstraps (--bs 10000) to generate a 95% confidence interval of ASE aFC. We then calculated Spearman's correlation between the aFC from *cis*-eQTL and those from ASE at the matched loci across all 34 tissues.

Trans-eQTL mapping and internal validation

We conducted an exploratory analysis of *trans*-eQTL in 12 tissues with over 150 individuals. To reduce the potential false positives in *trans*-eQTL mapping, we applied a stringent standard to filter genetic variants and genes[48]. We first calculated the mappabilities of genome-wide variants and the cross-mappabilities of genome-wide gene-pairs using crossmap (https://github.com/battle-lab/crossmap). We removed SNPs with 75 *k*-mer based mappability <1. We removed SNPs in repeat regions annotated by the UCSC RepeatMasker track[49] and kept SNPs with MAF >0.05 only for subsequent *trans*-eQTL analysis. For genes, we only considered those with an average mappability of ⩾0.8 for *trans*-eQTL mapping. For each gene, we only considered SNPs that did not fall in the same chromosome of target genes or within ±1Mb of its cross-mappable genes. We used a linear mixed model including a genomic relationship matrix in GCTA (v1.93.0) where the covariates were the same as in the *cis*-eQTL mapping.

For multiple testing correction, we first extracted the most significant *P*-value of the genome-wide level across all tested genes and multiplied by 10^6 as did in human GTEx[43], which was consistent with the assumed effective number of tests at the genome-wide level. We then corrected for the multiple testing at the gene level using the Benjamini-Hochberg method and defined genes with FDR < 0.05 as *trans*-eGene. For each *trans*-eGene, we further employed the Benjamini-Hochberg method to adjust the multiple testing for *P*-values and considered gene-SNP pairs with FDR < 0.05 as significant. We took the muscle that has the largest sample size (n=1,321) as an example to conduct an internal validation of *trans*-eQTLs by randomly dividing samples into two groups (Group1 and Group2) for *trans*-eQTL mapping separately.

Breed-sharing patterns of *cis*-eQTL

To explore how *cis*-eQTL are shared across breeds, we divided muscle samples into eight breed groups (i.e., Asian pigs, Duroc, Yorkshire, Landrace, Landrace×Yorkshire, Duroc×Luchuan, Duroc×Pietrain, and other crossbreeds), and then performed the *cis*-eQTL mapping as described above within each breed group separately using TensorQTL (v1.0.3)[50]. We calculated the π_1 statistic to measure the replication rate of *cis*-eQTL between breeds. In addition, to further explore the sharing pattern of *cis*-eQTL across breeds, we performed a meta-analysis of *cis*-eQTL across eight breed groups using MashR (v0.2-6)[42] and METASOFT (v2.0.1)[51]. We used the z-scores calculated from TensorQTL (i.e., slope/slope_se) of *cis*-eQTL of genes across all 34 tissues and 8 breed groups as input for MashR (v0.2-6). To fit the *mash* model, we first randomly selected 1,000,000 SNP-gene pairs that were tested in all breed groups as null inputs. We set missing z-scores as zero with a standard error of 10^6 *via* the *zero_Bhat_Shat_reset* = 10e6. We then obtained the estimate of effect size (i.e., the posterior mean) and the corresponding significance level (i.e., the local false sign rate, LFSR) from the *mash* function. To identify whether a *cis*-eQTL is active in a breed, we defined LFSR < 0.05 as the significance threshold, unless noted otherwise. To quantify the similarity of *cis*-eQTL effect sizes between two breeds, we calculated Spearman's correlation of effect size estimates of *cis*-eQTL significant (LFSR < 0.05) in at least one breed. Furthermore, we employed METASOFT (v2.0.1) to perform a meta-analysis of *cis*-eQTL across different breeds using summary statistics obtained from TensorQTL (v1.0.3) (i.e., slope and slope_se). We obtained estimates of meta-analytic effect sizes from a fixed effects model and M-values from a Markov Chain Monte Carlo (MCMC) method. The M-value represents the posterior probability that a *cis*-eQTL effect exists in a breed.

Breed and cell type interaction *cis*-eQTL

To detect *cis*-eQTL that may be specific to a particular breed, we performed the breed-interaction *cis*-eQTL (bieQTL) analysis. We first used imputed genotypes to estimate the ancestry composition of all RNA-Seq samples across tissues using ADMIXTURE (v1.3.0) with K = 5, representing Duroc, Landrace, Yorkshire, Asian pigs, and American pigs. We only considered 33 tissues that had breed ancestry with a median ancestry proportion > 0.1 for bieQTL mapping. We performed the bieQTL mapping using the following linear regression model with an interaction term between genotype and ancestry proportion, implemented in TensorQTL (v1.0.3), separately for each tissue-breed pair:

$$\boldsymbol{y} = \boldsymbol{A} + \boldsymbol{g} + \boldsymbol{b} + \boldsymbol{g} \times \boldsymbol{b} + \boldsymbol{e}$$

where $\boldsymbol{y}$ is the vector of gene expression values (i.e., the inverse normal transformed TMM), $\boldsymbol{A}$ represent the same covariates as in the regular *cis*-eQTL mapping, $\boldsymbol{g}$ is the genotype vector for a specific SNP, $\boldsymbol{b}$ is the proportion of a given ancestry (e.g., Duroc), $\boldsymbol{g} \times \boldsymbol{b}$ is the interaction term between genotype and ancestry proportion, and $\boldsymbol{e}$ is the residual error. We only considered SNPs within the *cis*-window (±1Mb

of TSS) of each gene for the bieQTL mapping. We filtered out SNPs with MAF < 0.1 in the top and/or bottom 50% of samples sorted by an ancestry proportion of interest (e.g., Doruc), using TensorQTL (v1.0.3) with option: --maf_threshold_interaction 0.1. We used eigenMT[52] in TensorQTL to correct for the multiple testing at the gene-level for the top nominal *P*-value of each gene. We then computed the genome-wide significance of genes using the Benjamini-Hochberg FDR correction on the eigenMT-corrected *P*-values. We defined genes that had at least one significant (i.e., FDR-corrected *P*-value < 0.01) bieQTL as bieGenes.

We detected cell type interaction *cis*-eQTL (cieQTL) in seven bulk tissues using the same approach as described above, where we replaced the ancestry composition with the estimated cell type composition obtained from CIBERSORTx based on single-cell RNA-Seq data from brain, lung and PBMC[21]. We considered cell types with a median enrichment percentage of > 0.1 within tissue for cieQTL mapping. We defined genes that had at least one significant (i.e., FDR-corrected *P*-value < 0.01) cieQTL as cieGenes.

ASE validation of interaction *cis*-eQTL

To validate the detected ieQTL, we estimated the effect size (aFC) of the top ieQTL of ieGenes from ASE data using the script *phaser_cis_var.py* in phASER (v1.1.1)[46]. Here, we only considered ieQTL that had nominally significant ASE (*P*-value < 0.05) data in more than 10 heterozygous individuals with more than 8 reads for a gene. To filter out samples with outlier ASE values, we applied Hampel's test, a median absolute deviation (MAD) based method, to the allelic imbalance (AI) ratio values ($|\frac{\text{Reference reads}}{\text{Total reads}} - 0.5|$) across samples[53,54]. We defined a sample as an outlier if it had $|\text{AI}_i - \text{median (AI)}| \geq 4.5 \times \text{MAD}$, where $\text{MAD} = \text{median}(|\text{AI}_i - \text{median(AI)}|)$ and AI_i is the allelic imbalance ratio value for i^{th} individual. We then calculated the Pearson's correlation between aFCs of an ASE locus and ancestry/cell type proportion estimates across the remaining samples within a tissue. We considered that an ieQTL was validated by ASE data if Pearson's correlation was nominally significant (at *P*-value < 0.05). For tissues with ⩾ 5 ieQTLs, we also calculated the π_1 statistics for significance (*P*-value) of Pearson's correlation across breeds/cell types in a given tissue using *qvalue* method[40] with a fixed lambda of 0.5 [54].

Sharing patterns of different molQTL

To explore the specificity/similarity of the five types of molQTL, we first generated approximate LD blocks (defined by Gabriel S et al. [55]) of SNPs across the entire genome for each tissue based on imputed genotypes using PLINK (v1.90) with parameters: --blocks no-pheno-req. For each tissue, we tested whether conditionally independent molQTL or ieQTL from different molecular phenotypes were located in the same LD block. We defined a molQTL that is unique to a molecular phenotype if it was not located in the same LD block with other types of molQTL.

Sequence conservation of *cis*-eQTL

To understand the evolutionary sequence conservation of the different types of *cis*-eQTL, we downloaded PhastCons scores of 100 vertebrate species from UCSC (http://hgdownload.cse.ucsc.edu/goldenpath/hg38/phastCons100way/hg38.100way.phastCons/). We first converted the Wiggle files of PhastCons scores to bed files using the BEDOPS tool (v2.4.40)[56], and then lifted over from human genome 38 (h38) to Sscrofa11.1 using UCSC's LiftOver tool[57]. We used the mean phastCons scores of sequences within a gene to represent the PhastCons score of this gene. We only considered genes that had a minimum matched the length of sequences ⩾ 80% of the gene's length in LiftOver.

Detecting *cis*-eQTL with opposite effects between tissues

To understand the tissue-specific effects of *cis*-eQTL, we examined *cis*-eQTL with opposite effects between any pair of tissues[58]. For a given tissue pair, if the top *cis*-eQTL of a given eGene were identical in the two tissues or in high LD ($r^2 > 0.8$), we designated the top *cis*-eQTL as a multi-eQTL and the eGene as a multi-eGene. We then defined the multi-eQTL whose estimated effects on the multi-eGene showed the opposite direction in the two tissues as opp-multi-eQTL and the target multi-eGene of the opp-multi-eQTL as an opp-multi-eGene.

QTL mapping for DNA methylation (meQTL) in muscle

We conducted QTL mapping for methylation level at each CpG locus with a coverage of at least five reads in muscle, where we had 101 WGBS samples. We called SNPs from the WGBS data using *BisulfiteGenotyper* in the Bis-SNP package (v1.0.1, and dbSNP build 150) with parameters: -nt 4 -stand_call_conf 10 -mmq 30 -mbq 17 -out_modes EMIT_ALL_SITES[59]. After quality control using PLINK2[31] (MAF ⩾ 0.05, DR^2 ⩾ 0.8, and Hardy Weinberg equilibrium, HWE ⩾ 1e-5), 7,410,484 SNPs remained for subsequent analysis. For methylation, we excluded CpG sites with missing rate ⩾ 10% across samples. To improve the efficiency of QTL mapping, we only considered CpG loci with a deviation of methylation levels > 0.3 and a standard deviation > 0.1, yielding 18,023,521 CpG sites on 18 autosomes. We applied the rank-based inverse normal transformation to the methylation levels across samples for each CpG site and predicted 10 hidden factors using PEER (v1.3)[10]. We mapped SNPs within 1Mb around a CpG site to identify the significant SNP-CpG pairs using FastQTL (v2.184)[60] at the threshold of *P*-value < 1e-8, considering five genotype PCs, ten PEER factors, bioproject, sex and sequencing platforms as covariates. On average, we tested 6,800 SNPs for each CpG site. We removed significant meQTL that overlapped with any CpG sites (either C or G).

Functional enrichment analysis of molQTL

To investigate molecular mechanisms underlying the regulatory variants detected above, we examined multi-layer biological data as features, including SNPs annotated by SnpEff v.4.3[61], sequence ontology (e.g., intron and UTR), 15 chromatin states from 14 different tissues[6], and the DNA methylation features (i.e., HMR, ASM and meQTL) identified above. Similar to the human GTEx[43], we employed a Bayesian hierarchical model and an expectation–maximization (EM) algorithm with the *-est* option of TORUS[62] to explore whether the five types of identified molQTL were enriched for variants within these biological features. We further employed SuSiE-inf (v1.2)[63] to perform fine mapping for all five types of molQTL. We selected molQTL with the highest posterior inclusion probability to represent the causality score of each molecular phenotype[64]. We then divided molQTL into three causality groups (high: top 1/3, medium: 1/3-2/3, and low: bottom 1/3) based on the rank of causality scores. Furthermore, we conducted the enrichment analysis of each molQTL bin in sequence ontology and chromatin states. Following the human GTEx approach[43], we examined whether the independent *cis*-eVariants were significantly enriched in the same TAD with their target eGenes. Briefly, in a tissue, we first randomly selected 1M variants from the *cis*-window of genes being tested for *cis*-eQTL as a null file. We then kept all the top variants in each *cis*-eQTL as an eQTL file. We accessed the degree of enrichment of eQTL in the same TAD with their target genes using the following formula: SameTAD ~ eQTL + |TSSdistance| + eQTL*|TSSdistance|, where SameTAD is an indicator of whether a variant-gene pair resides within the same TAD, eQTL is an indicator of whether the variant-gene pair is an eQTL or null, and |TSSdistance| is the absolute distance between the variant and the TSS of the gene. Moreover, to predict genetic variants that alter transcription factor binding sites (TFBS), we applied a custom script[65] with TFBS models from

the JASPAR (CORE 2018)[66], HOCOMOCO (v10)[67], and TRANSFAC (v3.2 public)[68] databases. The TFBS models were represented as position weight matrices (PWM) and were derived from published collections of experimentally defined eukaryotic TFBS. We used the vertebrate PWM and kept only a candidate regulatory variant if the gene and the transcription factor, both impacted by the *cis*-eQTL, were both expressed in the same tissue.

Heritability of complex traits mediated by molQTL

To explore if randomly chosen MAF-matched SNPs could explain similar amounts of heritability as the molQTL for complex traits in pigs, we applied two approaches, including LDAK (using individual genotype and phenotype data)[69] and MESC (using GWAS summary data)[70] to partition heritability of 16 different complex traits with large sample sizes in pigs (n=4,127~4,383). For LDAK analysis, we used a mixed linear model with multiple genetic components to partition the heritability of a complex trait into three different SNP sets (i.e., independent molQTL, random MAF-matched SNPs and the remaining SNPs): $y = Xa + Zg_{molQTL} + Zg_{random} + Zg_{remaining} + e$, where y is the vector of phenotypes of the target complex trait, X is the covariates matrix, a is the effects of covariates, Z is the design matrix that allocates phenotypes to genetic values, $g_{molQTL} \sim N(0, G_{molQTL}\sigma^2_{g_{molQTL}})$, $g_{random} \sim N(0, G_{random}\sigma^2_{g_{random}})$, and $g_{remaining} \sim N(0, G_{remaining}\sigma^2_{g_{remaining}})$ are the polygenic effects of conditionally independent molQTL, random MAF-matched SNPs, and the remaining SNPs, respectively, where G_{molQTL} , G_{random} and $G_{remaining}$ are GRMs calculated based on genotypes of conditionally independent molQTL, random MAF-matched SNPs, and the remaining SNPs, respectively. $\sigma^2_{g_{molQTL}}$, $\sigma^2_{g_{random}}$ and $\sigma^2_{g_{remaining}}$ are genetic variance explained by conditionally independent molQTL, random MAF-matched SNPs, and the remaining SNPs, respectively. e is the residual. For MESC analysis, we employed a previous approach[70] to compute the heritability of complex traits mediated by gene expression that were regulated by independent molQTL or random MAF-matched SNPs. In brief, the contribution of a SNP set to the total heritability of a complex trait can be calculated by $h^2_{med-SNP} = \frac{\mathcal{L}_{sub}}{\mathcal{L}_{total}} * h^2_{med}$, where $h^2_{med-SNP}$ is the heritability of a complex trait explained by SNPs of interest (conditionally independent molQTL or random MAF-matched SNPs), h^2_{med} is the expression-mediated heritability by *cis*-component of all genes, $\mathcal{L}_{total}$ is the sum of expression scores of all tested SNPs, $\mathcal{L}_{sub}$ is the sum of expression scores of SNPs of interest. The expression score of a SNP that was obtained from MESC represents the contribution of SNP to the total expression *cis*-h^2 of genes[70].

Comparative transcriptome between pigs and humans

We compared gene expression and its genetic regulation between pigs and humans in 17 common tissues, including adipose, artery, blood, colon, frontal cortex, heart, hypothalamus, ileum, kidney, liver, lung, muscle, ovary, pituitary, spleen, testis, and uterus. We downloaded gene expression and *cis*-eQTL data of 15,044 samples from the Human GTEx web portal (v8, https://gtexportal.org/home/) and considered genes with TPM > 0.1 as expressed. We divided genes into the following three groups based on the orthologous annotation from Ensembl (v100): one-to-one orthologous genes (n= 15,944), complex orthologous genes (n=2,395 in pigs, and n=2,605 in humans, 1-to-many, many-to-1, and many-to-many), and non-orthologous genes (n=13,569 in pigs, and n=37,651 in humans). We integrated the gene expression matrices from pigs and humans (5,173 and 15,044 samples in pigs and humans, respectively) using Seurat (v3.0), while correcting for unknown batch effects. We then visualized the divergence in gene expression between pig and human samples using *t*-SNE[71]. We detected tissue-specific genes for

humans using the same method as described above for pigs. Within each tissue, we divided genes into four groups by comparing eGenes between pigs and humans: 1) eGenes shared by both species (Both), 2) eGenes only identified for humans (Human-specific), 3) eGenes only identified for pigs (Pig-specific), 4) non-eGenes in both species (Neither). To test the conservation of eGenes between pigs and humans, we did the Fisher exact test and obtained odd ratio (OR) values. To investigate the difference between these four gene groups in sequence conservation between pigs and humans, we used PhastCons scores of 100 vertebrate genomes, as described above. To explore their difference in the tolerance to loss of function mutations (LOF), we obtained the LOEUF scores from gnomAD (v2.1.1)[72]. We collected the *cis*-heritability estimations of human tissues from the previous study[73]. We obtained orthologous variants between pigs and humans using LiftOver[57], among which 112 SNPs were *cis*-eQTL in at least one tissue in humans. In addition, we compared the gene expression and their genetic regulator effects for 14,583 one-to-one orthologous genes across pigs, cattle and humans.

Supplementary Figures and Legends

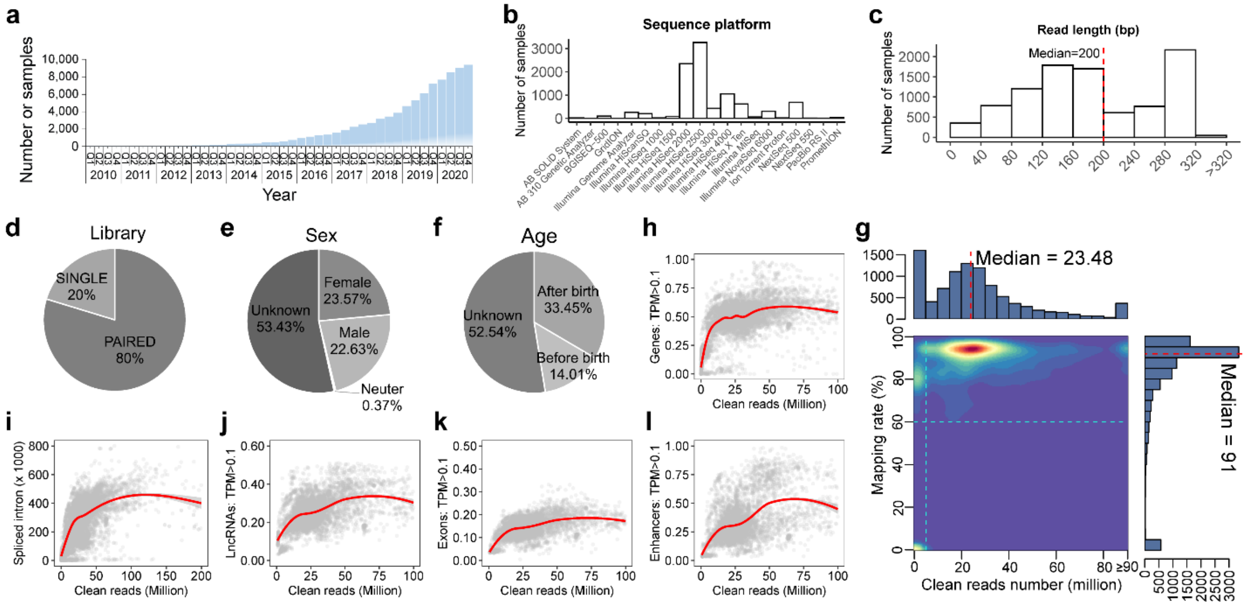

Supplementary Fig. 1 | Summary of publicly available RNA-Seq data in the pilot PigGTEx. a, Number of publicly available RNA-Seq samples over the past years from 2010 to 2020. Q1-Q4 represents the four quarters of a year. **b-g,** Distribution of 9,530 RNA-Seq samples regarding sequencing platforms (**b**), read length (**c**) sequencing strategies (single or paired-end) (**d**), sex (**e**), age (**f**), mapping rates and number of clean reads (**g**). **h-l,** Number of expressed genes (Transcripts Per Million, TPM > 0.1) (**h**), spliced introns (**i**), lncRNAs (TPM > 0.1) (**j**), exons (TPM > 0.1) (**k**) and enhancers (TPM > 0.1) (**l**) increase with the increasing number of clean reads across 9,530 samples. The smoothed lines are fitted by a local polynomial regression model using the *geom_smooth* function from ggplot2 (v3.3.2) in R (v4.0.2).

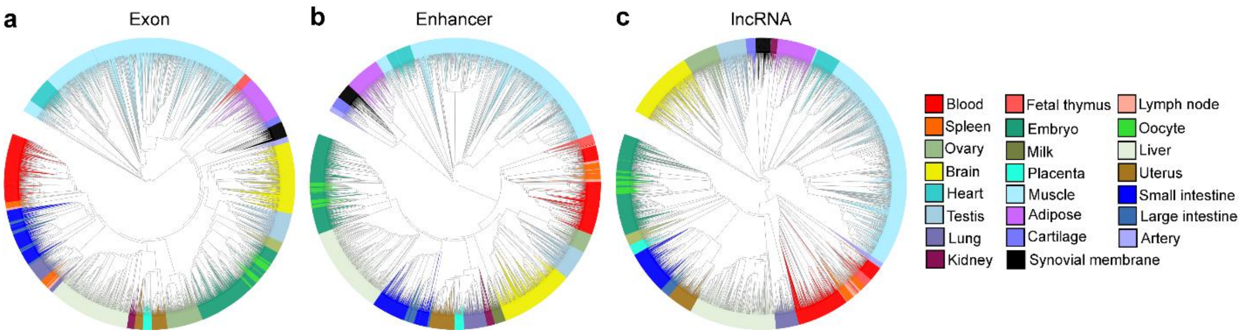

Supplementary Fig. 2 | Phylogeny clustering of 7,095 RNA-Seq samples. **a,** Sample clustering based on normalized expression levels (Transcripts Per Million, TPM) of 9,500 highly variable exons, defined as the top 20% of exons with the largest standard deviation of TPM across samples. **b,** Sample clustering based on 7,500 highly variable enhancers, defined as the top 5% of enhancers with the largest standard deviation of TPM across samples. **c,** Sample clustering based on 9,500 highly variable lncRNAs, defined as the top 20% of lncRNAs with the largest standard deviation of TPM across samples.

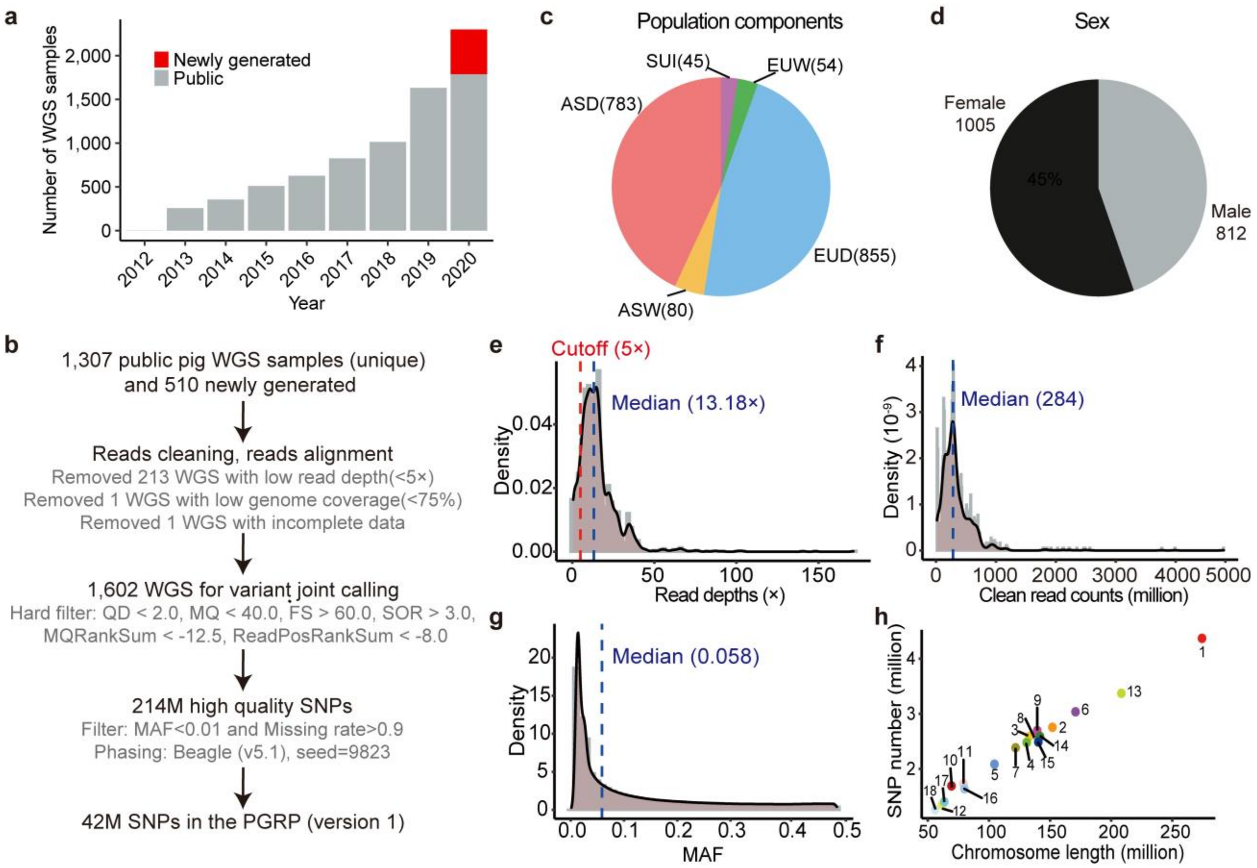

Supplementary Fig. 3 | Summary of whole-genome sequence (WGS) data used in the pig genomics reference panel (PGRP). a, Number of publicly available WGS samples over the past years and newly generated samples in this study. **b,** The whole pipeline utilized to process 1,817 WGS samples for building the pig genomics reference panel (PGRP). **c,** Number of samples in the major pig populations, including Suidae but not Sus scrofa (SUI), European wild pig (EUW), European domestic pig (EUD), Asian wild pig (ASW) and Asian domestic pig (ASD). **d,** Sex distribution of all 1,817 WGS samples. **e,f,** Density plot of read depths (**e**) and clean reads (**f**) across all 1,817 samples. **g,** Density plot of minor allele frequency (MAF) of all 42 million SNPs in the PGRP. **h,** Number of SNPs increases with the increasing length of 18 chromosomes.

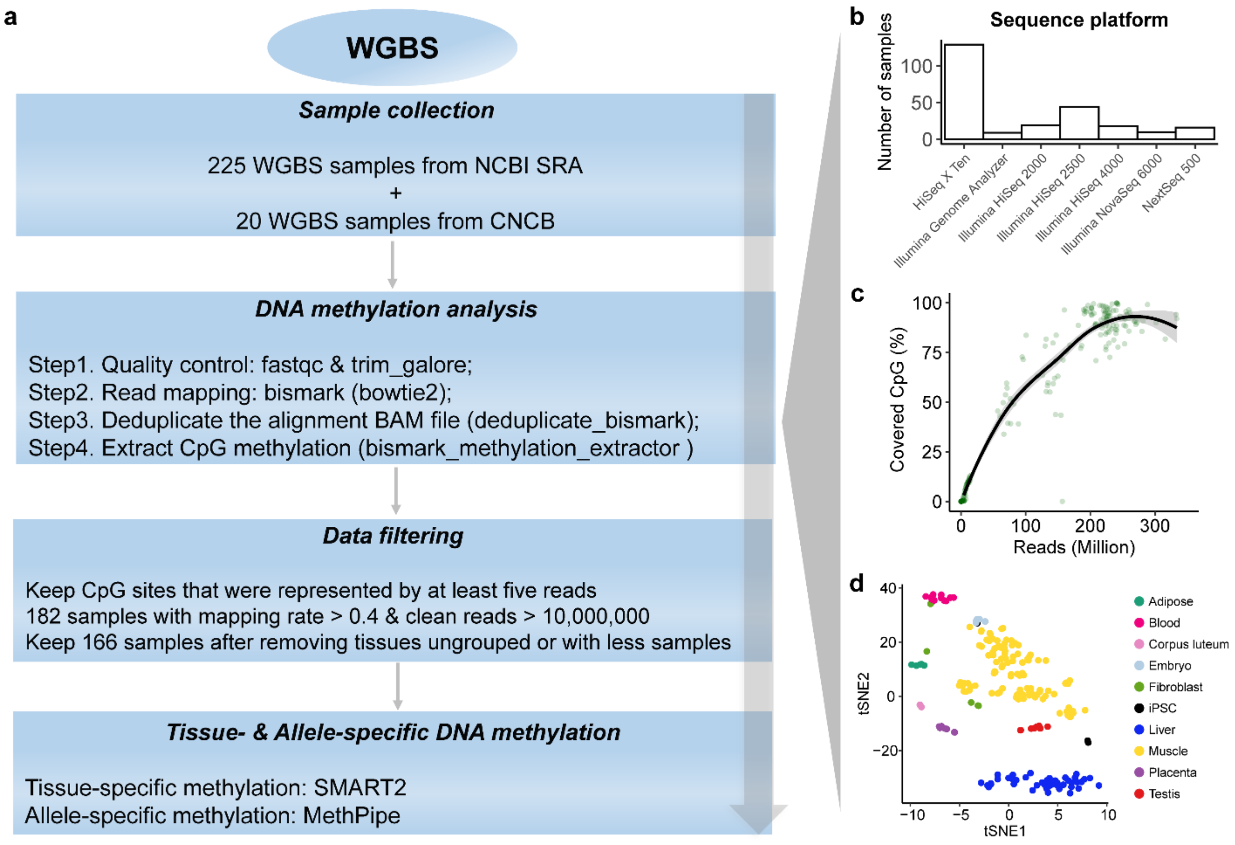

Supplementary Fig. 4 | Summary of 245 whole-genome bisulfite sequencing (WGBS) data in this study. a, The whole pipeline utilized to process 245 WGBS samples. **b,** Sequence platform distribution of 245 WGBS samples. **c,** The percentage of covered CpGs (read depth ≥ 5×) across the genome increases rapidly with the increasing number of reads used for methylation extraction. **d,** Sample (n=182) clustering using t-distributed stochastic neighbor embedding (t-SNE) coordinates based on DNA methylation levels of 9,347 CpG sites (coverage ≥ 5×).

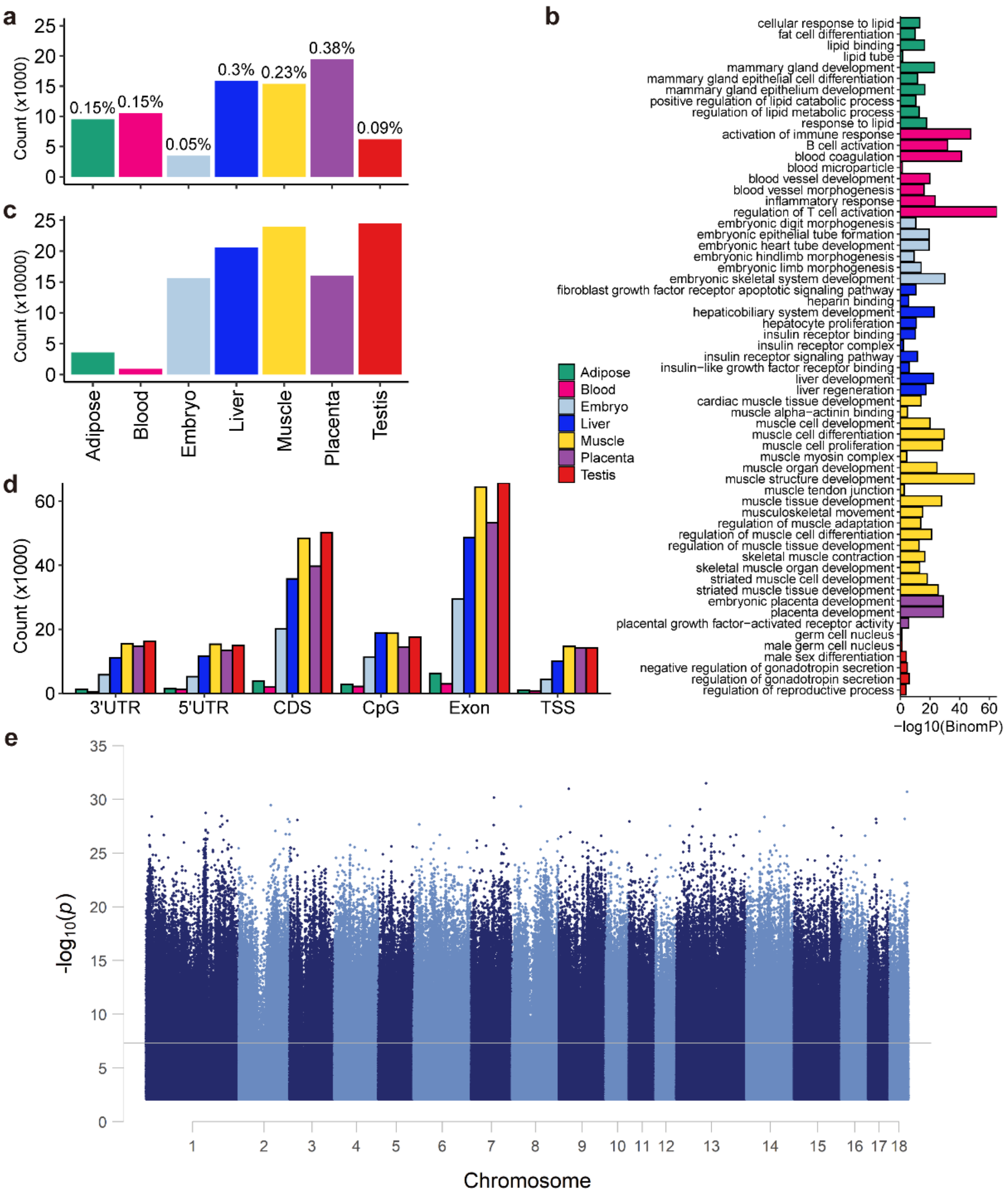

Supplementary Fig. 5 | Characteristics of DNA methylation across tissues. a, Counts of hypomethylated regions (HMRs) detected across seven tissues. The numbers above the bars represent the percentage of the whole genome covered by HMRs. **b,** Functional enrichment results of tissue-specific HMRs in respective tissues. The binomial *P* value (BinomP) is obtained from the binomial test implemented in GREAT. The functions of tissue-specific HMRs matched the known biological attributes of the respective tissues. **c,** Counts of allele-specific methylation (ASM) loci detected across tissues. **d,** Distribution of ASM loci along different sequence features. UTR: untranslated region; CDS: coding sequence; TSS: transcriptional start site. **e,** Methylation quantitative trait loci (meQTL) detected in muscle (n=101), where we detected 3,756,288 meQTL for 878,808 CpG sites.

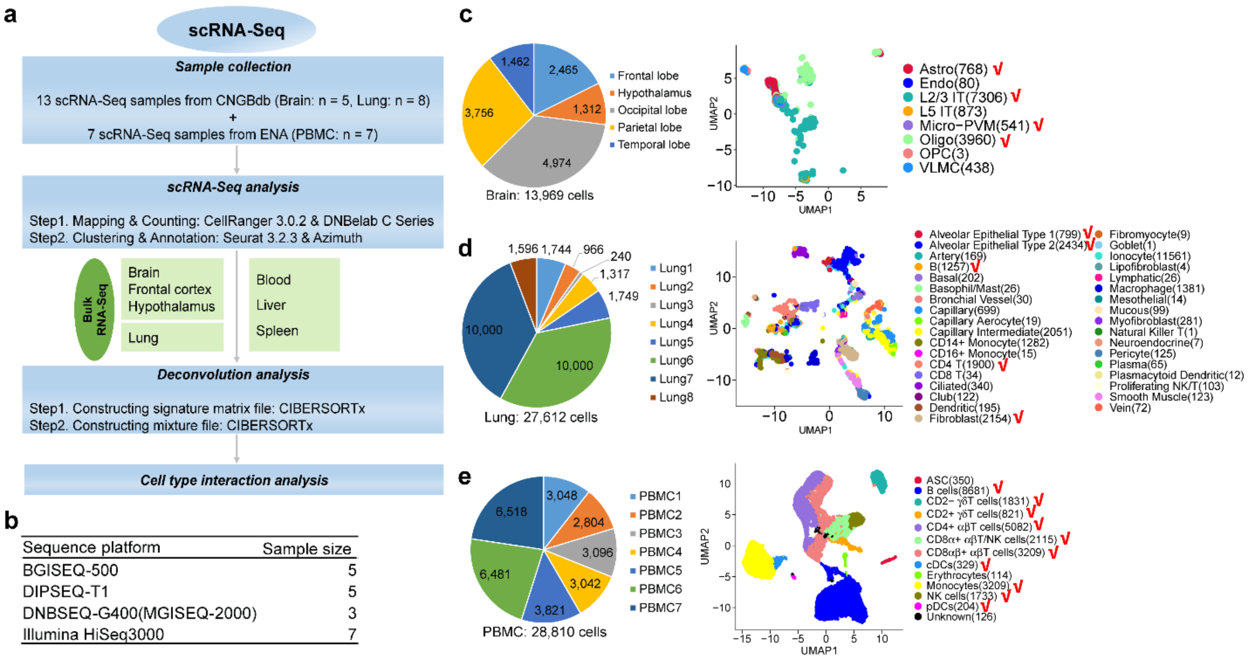

Supplementary Fig. 6 | Summary of single-cell RNA-Seq data analyzed in this study. a, Pipeline utilized for processing scRNA-Seq and cell-type deconvolution. The single-cell RNA-Seq data consisted of 13,969 cells in the brain (n=5), 27,612 cells in the lung (n=8), and 28,810 peripheral blood mononuclear cells (PBMC, n=7). **b,** Sequencing platform distribution of all 20 scRNA-Seq samples. **c-e,** Number of cells detected in different samples (left) from the brain (**c**), lung (**d**), and peripheral blood mononuclear cell (PBMC) (**e**), and two-dimensional uniform manifold approximation and projection (UMAP) visualization of single cells classified into different cell types correspondingly (right). We respectively selected 4, 5 and 10 major cell types (highlighted by a red check mark) in the brain, lung and PBMC, respectively, and used the single cell type expression for the cell type deconvolution analysis of bulk RNA-Seq samples from relevant tissues.

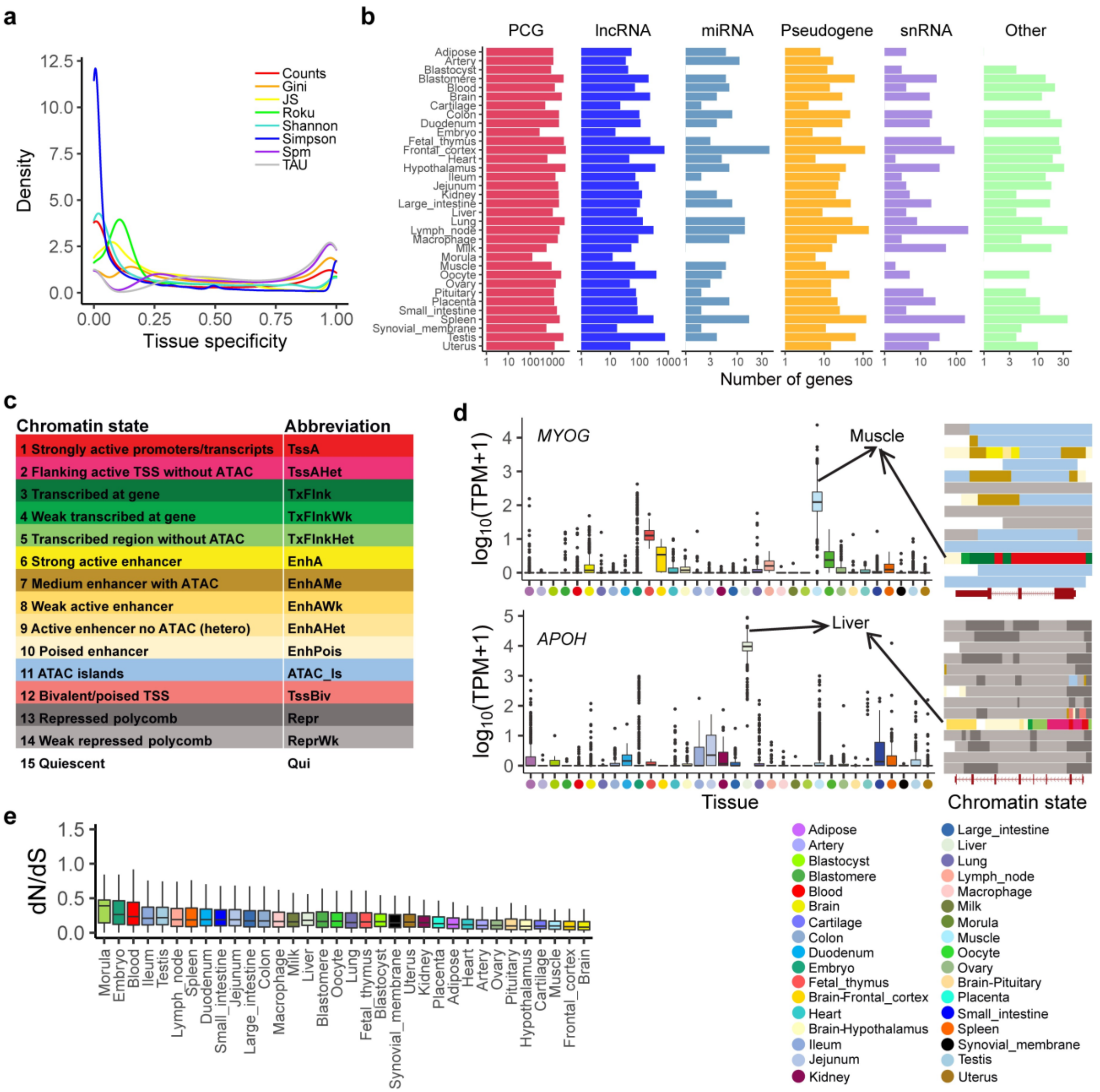

Supplementary Fig. 7 | Features of the pig gene expression atlas. a, Tissue specificity of 31,908 annotated genes (Ensembl v100) obtained from 7,095 RNA-Seq samples across 34 tissues using eight different measurements. **b,** Number of tissue-specific genes detected in each of 34 tissues across six gene types. Other means the rest of gene types. **c,** Definitions and abbreviations of 15 chromatin states detected previously from 14 pig tissues[6], including stomach, spleen, muscle, lung, liver, jejunum, ileum, hypothalamus, duodenum, cortex, colon, cerebellum, cecum, and adipose. **d,** Expression profiles of *MYOG* and *APOH* across 34 tissues (left). Chromatin states (right) around *MYOG* and *APOH* in 14 tissues. TPM: transcripts per million. Color codes for tissues are shown at the bottom right of the panel. **e,** The dN/dS ratio obtained between pigs and humans for tissue-specific genes across 34 tissues.

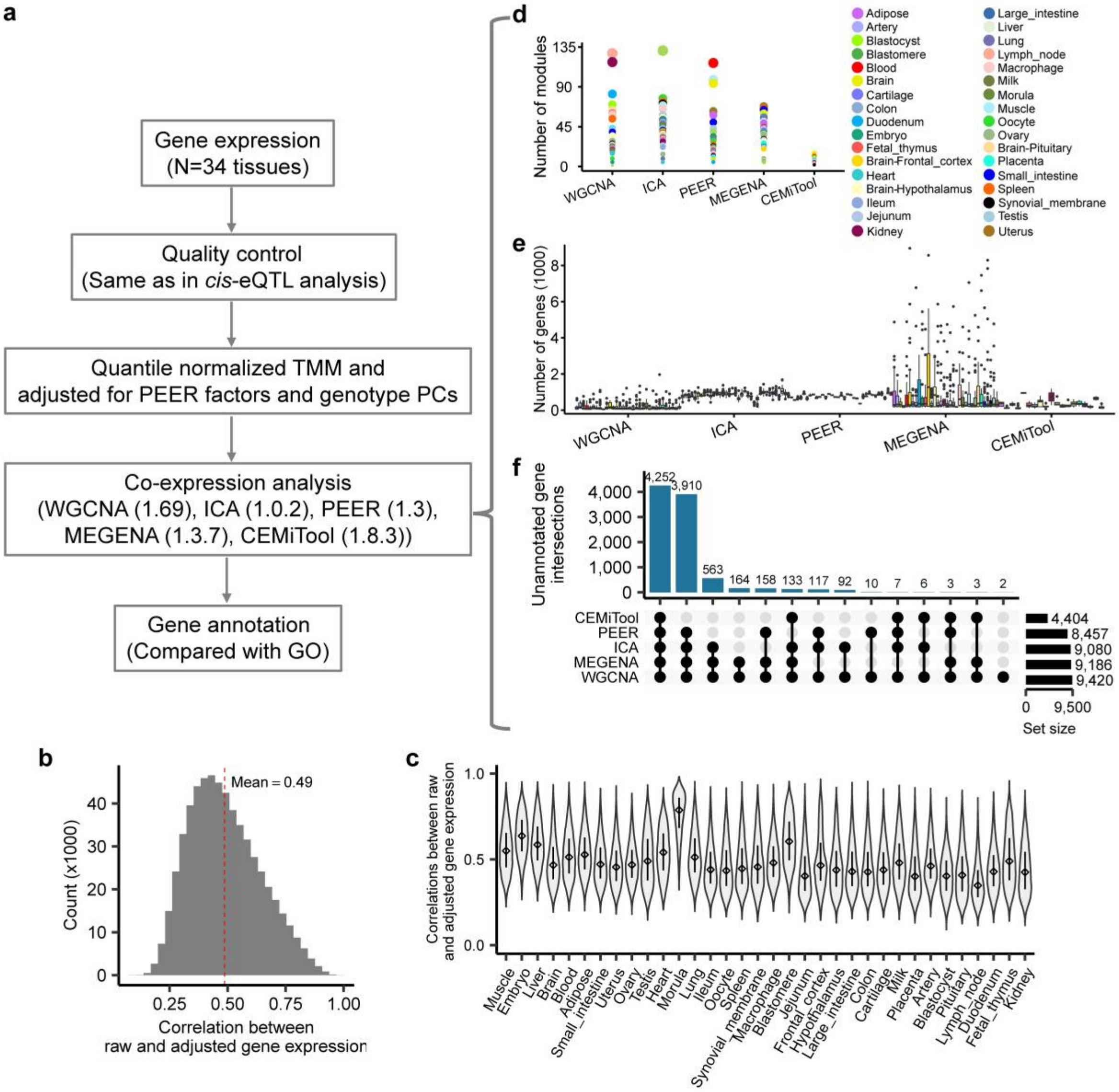

Supplementary Fig. 8 | Gene co-expression analysis. a, Pipeline for gene co-expression analysis in 5,457 RNA-Seq samples from 34 tissues using five different methods, followed by gene annotation with Gene Ontology database and eQTL mapping for gene co-expression modules. TMM: Trimmed Mean of M-value normalized expression levels; PEER[10]: Probabilistic Estimation of Expression Residuals; PC: principal components; WGCNA[8]: weighted correlation network analysis; ICA[9]: independent component analysis; MEGENA[11]: Multiscale Embedded Gene Co-expression Network Analysis; CEMiTool[12]: Co-Expression Modules identification Tool. **b,** Histogram of correlations between raw and adjusted gene expression levels across 34 tissues, where gene expression was adjusted for PEER factors and genotype PCs. **c,** Comparison of correlations between raw and adjusted expression levels among 34 tissues. **d,** Number of modules detected in 34 tissues by different methods. Color codes for tissues are shown at the bottom of the panel. **e,** Number of genes in all co-expression modules determined in 34 tissues by different methods. **f,** Number of unannotated genes shared between different methods. Unannotated genes represent those genes that had no functional annotation in the Gene Ontology (GO) database.

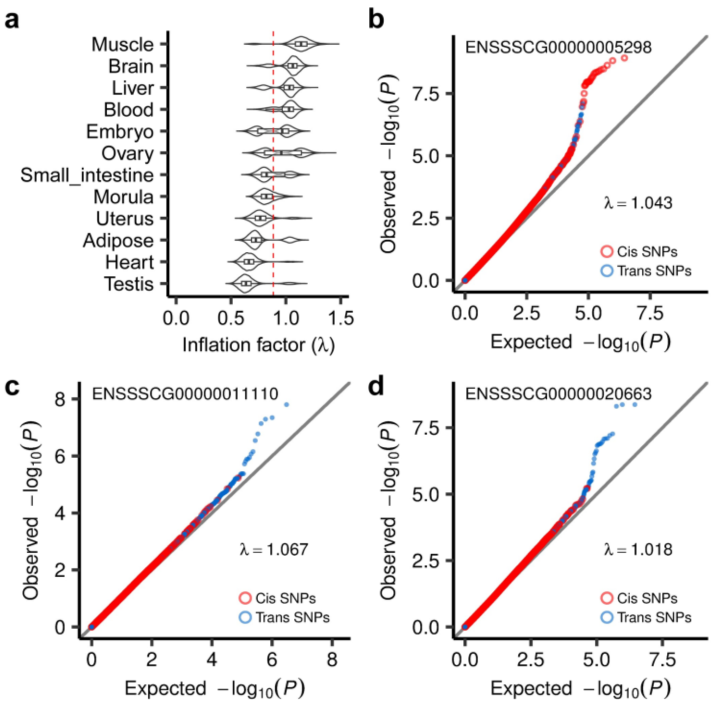

Supplementary Fig. 9 | Genomic control for molQTL mapping. a, Distribution of the genomic control inflation factor (λ) for all tested genes in 12 tissues with over 150 samples. The red line represents the mean λ (0.89) across 12 tissues. **b-d,** Quantile-Quantile (QQ) plots of genome-wide associations for *C9orf131* (*ENSSSCG00000005298*) in the muscle, *CCDC3* (*ENSSSCG00000011110*) in the liver, and *KMT2C* (*ENSSSCG00000020663*) in the blood.

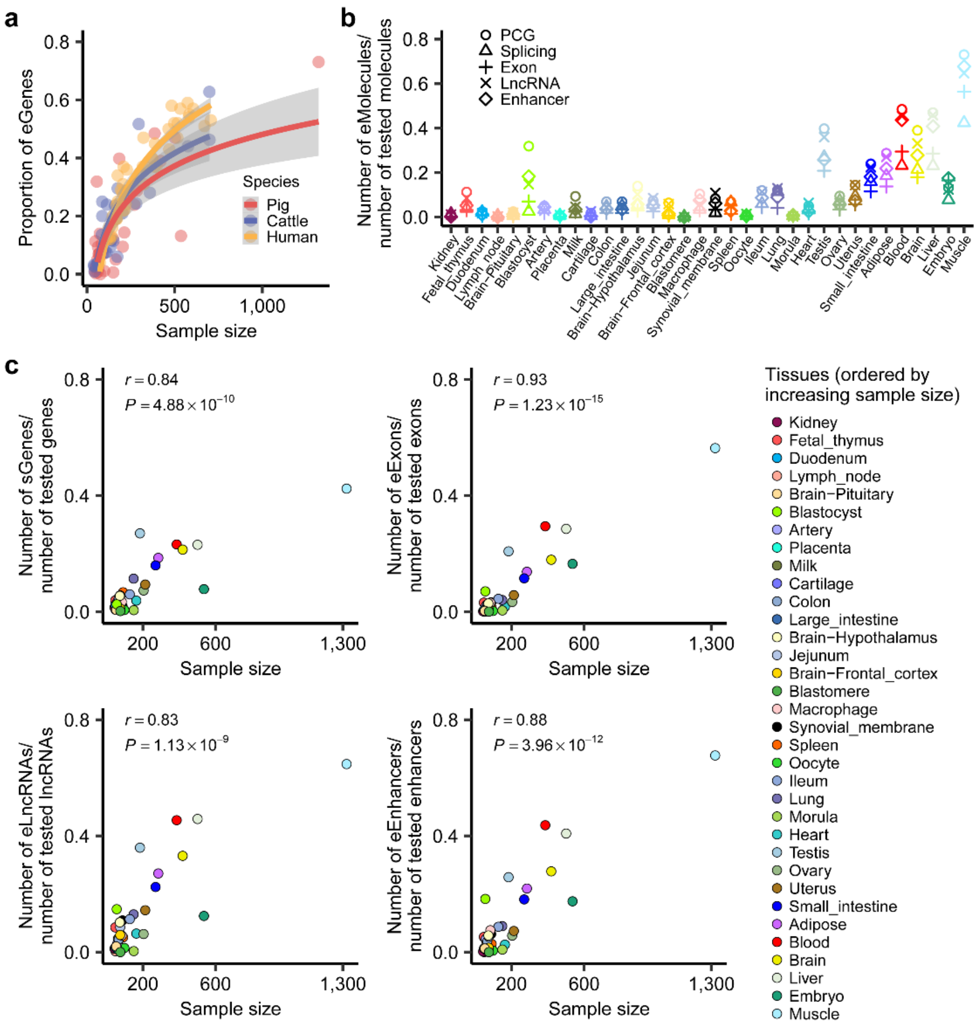

Supplementary Fig. 10 | Relationship between tissue sample size and the proportion of molecular phenotypes with detectable molecular quantitative trait loci (molQTL). a, Relationship between the proportion of eGenes and the sample size for the tissue in PigGTEx, CattleGTEx, and HumanGTEx (v8). The curves were fitted with y ~ log(x) by the *geom_smooth* function in ggplot2. **b,** Proportion of eMolecules in each tissue for five types of molQTLs in each of 34 tissues in pigs. Tissues are ordered by increasing sample sizes. **c,** Pearson's correlation (*r*) between the proportion of detectable eMolecules and sample size across 34 tissues. *P* values are obtained based on Pearson's correlation test.

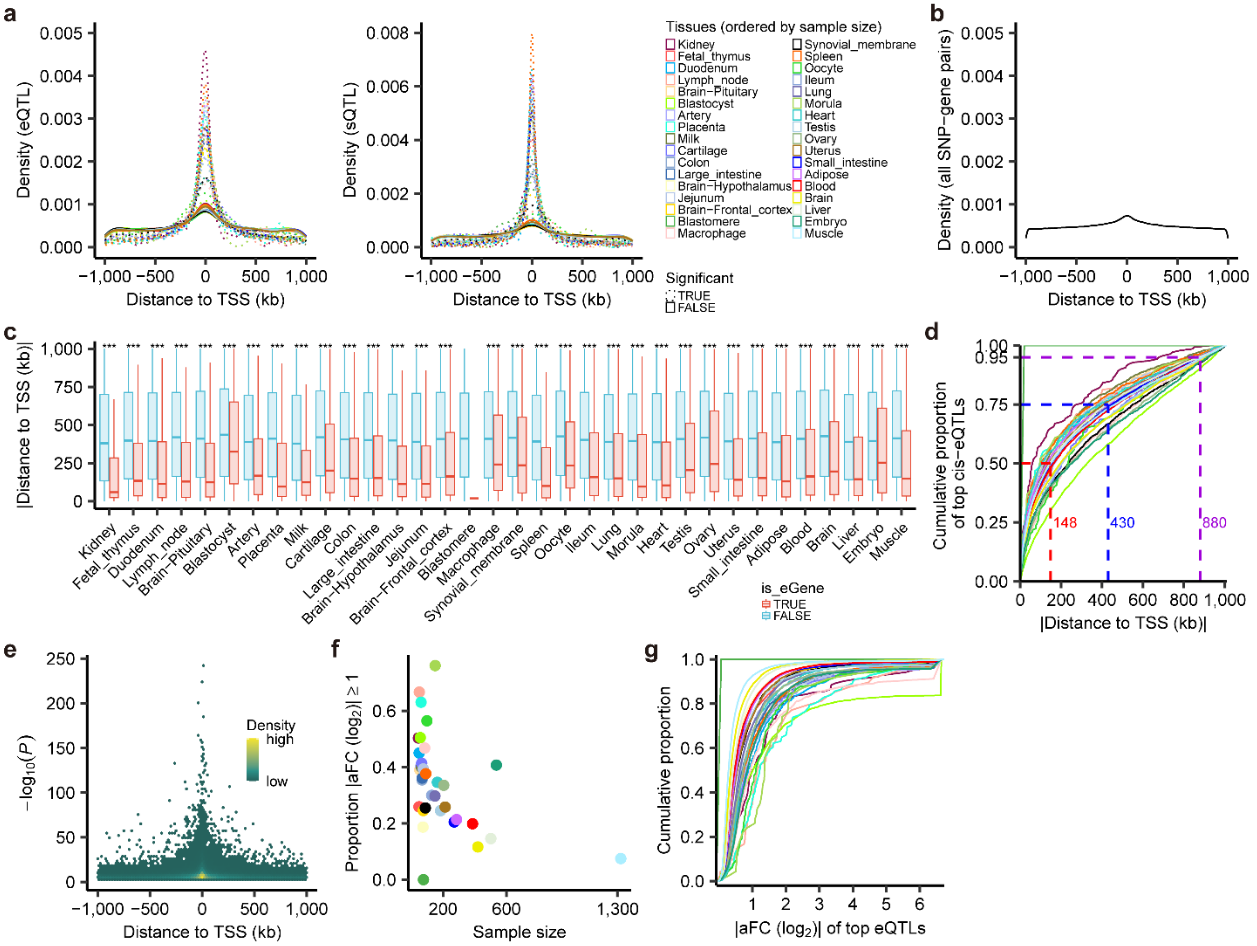

Supplementary Fig. 11 | Spatial distribution and effect sizes of molecular QTLs (molQTL). a, shows the distribution of top *cis*-QTL around (± 1Mb) transcript start site (TSS) of e/sGenes and the top associated variants for non-e/sGenes across 34 tissues. **b,** shows all tested gene-variant pairs. **c,** Box plot showing the distances of the most significant associated variants to TSS of the target genes for eGenes and non-eGenes. *** represents $P < 0.001$ based on the two-sided Wilcoxon rank-sum test. **d,** Cumulative proportion of distance to TSS of target genes for top *cis*-eQTL across 34 tissues. **e,** Distribution of significant levels ($-\log_{10}$ adjusted nominal P-value) of *cis*-eQTL around (± 1Mb) TSS of protein-coding genes across 34 tissues. **f,** Relationship between tissue sample size and proportions of detectable *cis*-eQTL with over two-fold effect (i.e., $|\text{aFC}(\log_2)| \geq 1$) on gene expression. The aFC is for allelic fold change. **g,** Cumulative proportion of effect sizes for top *cis*-eQTL across tissues. The meanings of colors of curved lines (**d**, **g**) and dots (**f**) are the same as the color key in panel (**a**).

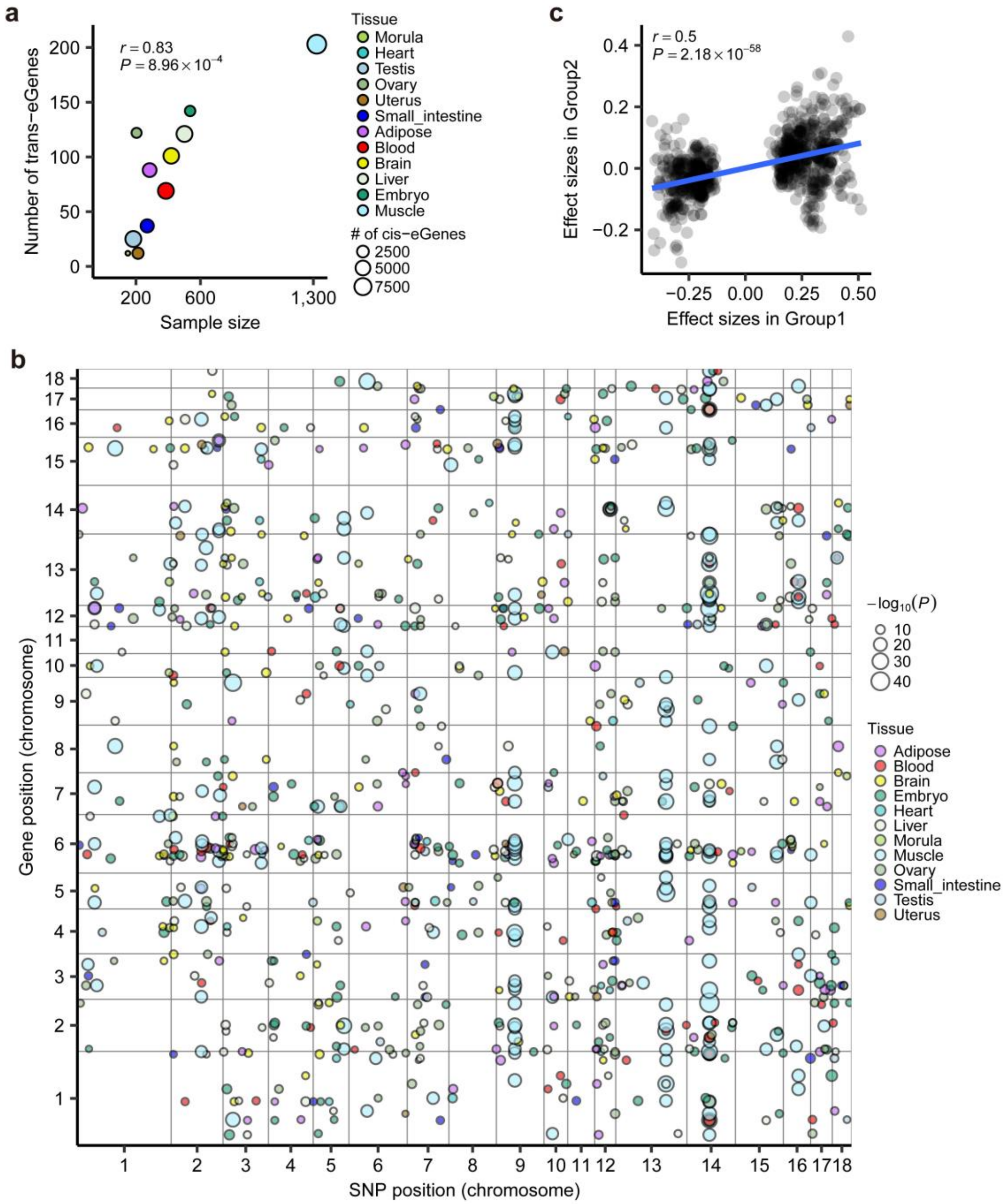

Supplementary Fig. 12 | Locations and internal validation of *trans*-eQTLs. a, Pearson's correlation (r) between the number of *trans*-eGenes (FDR < 0.05) and sample size across 12 tested tissues with sample size >150. **b,** Locations and association P-values (-log10 scale) of the most significant *trans*-eQTL (FDR < 0.05) for each *trans*-eGene in 12 tested tissues. **c,** Pearson's correlation of effect sizes of 925 significant (FDR < 0.05) *trans*-SNP-gene pairs in muscle in Group1 and those of matched SNPs in Group2, where we conducted the internal validation of *trans*-eQTL by randomly and evenly dividing samples into two groups (Group1 and Group2). P values were obtained based on the Pearson's correlation test.

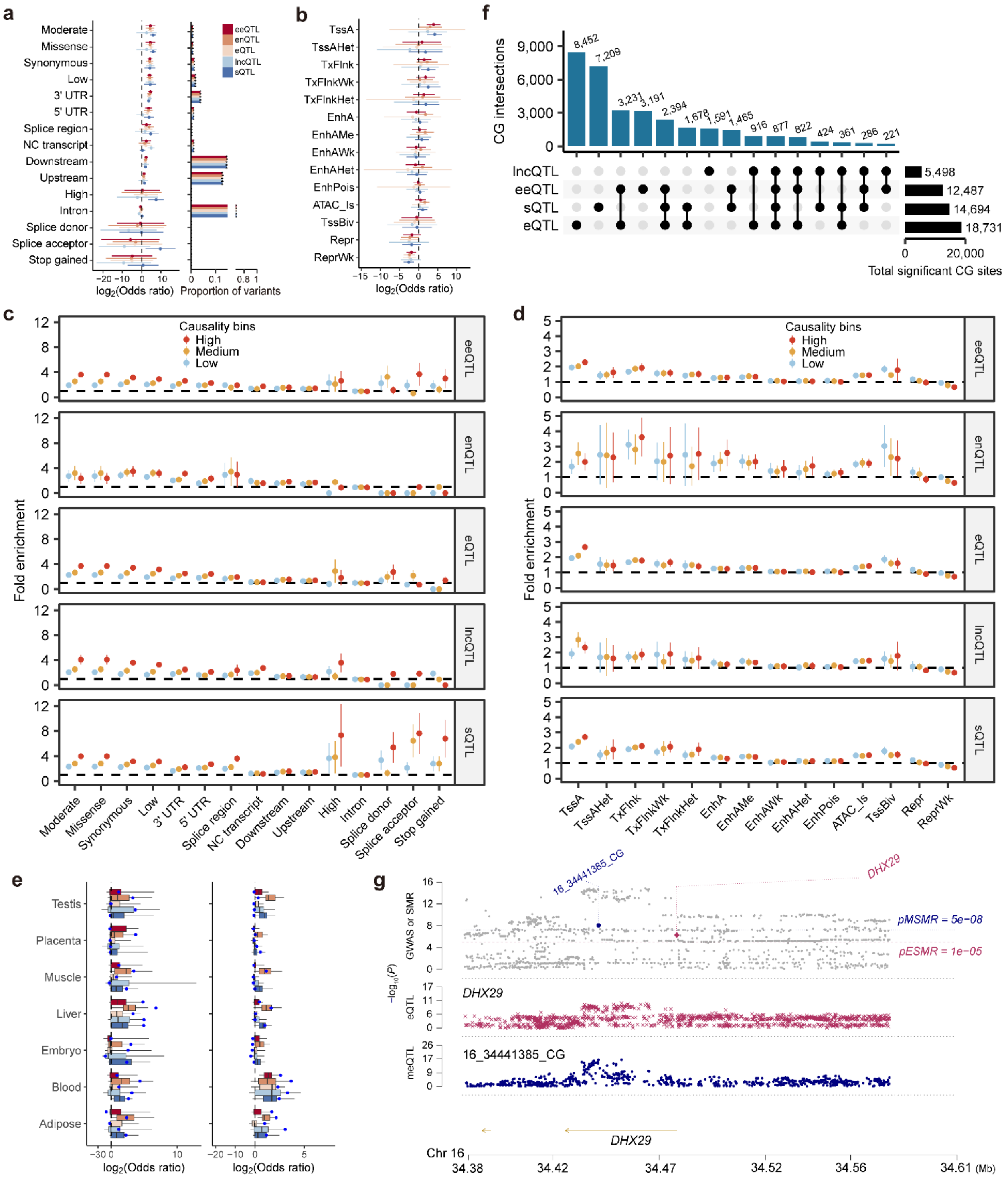

Supplementary Fig. 13 | Functional characterization of *cis*-QTLs. a,b, Enrichment (odds ratio±s.d.) of five types of molQTL in sequence ontology (**a**) and 14 chromatin states[6] (**b**). **c,d,** Fold enrichment (mean±s.d.) of fine-mapped molQTL in each causality bin for the intersection with sequence ontologies (**c**) and chromatin states (**d**), where molQTL were divided into three causality groups (high: top 1/3, medium: 1/3-2/3, and low: bottom 1/3) based on the rank of causality scores calculated using SuSiE-inf (v1.2). **e,** Enrichment of five molQTL types in DNA hypomethylated regions (HMR, left) and allele-

specific methylation loci (ASM, right) across seven tissues that have both DNA methylation and *cis*-eQTL data. The color key of molQTL is the same as in (**a**). Blue dots represent enrichments from methylation-molQTL matching tissues. **f,** Number of methylation QTL (meQTL) associated with different types of molecular QTL (molQTL) in muscle. The association of meQTL and molQTL was obtained using the summary-data based Mendelian randomization (SRM) test at $P_{SMR} < 5\times10^{-8}$ and $P_{HEIDI} \geq 0.05$. **g,** The summary data-based Mendelian randomization (SMR) results of methylation QTL (meQTL), *cis*-eQTL and GWAS of loin muscle area around *DHX29* gene in muscle. The top plot shows $-\log_{10}(P)$ of SNPs from GWAS. The red diamond and blue circle represent $-\log_{10}(P)$ from the SMR test for associations with gene expression and DNA methylation, respectively. The middle plot shows *cis*-eQTL results of *DHX29*. The bottom plot shows meQTL results at the CpG locus (16_34441285_CG).

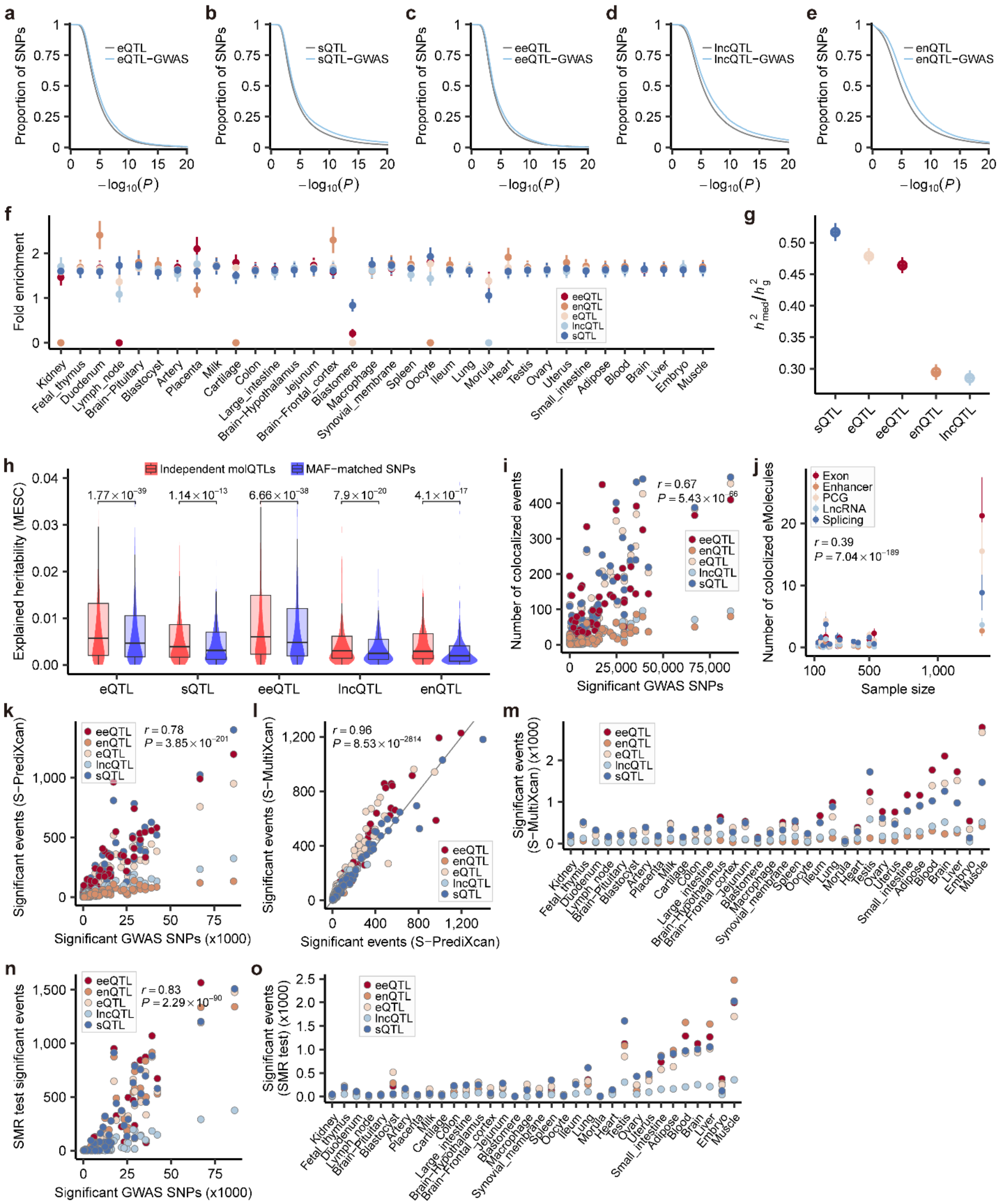

Supplementary Fig. 14 | Enrichments and colocalizations of *cis*-QTL and GWAS loci. a-f, Molecular QTL (molQTL) enrichment among GWAS loci ($P < 5\times10^{-8}$). The proportion of genetic variants associated with at least one protein-coding gene (**a**), splicing event (**b**), exon (**c**), lncRNA (**d**) and enhancer (**e**) in at least one tissue at different *P*-value cutoffs. **f,** Fold-enrichment (mean±95% CI) of

GWAS variants ($P \leq 0.05$) with different molQTL measured by QTLEnrich (v2) across tissues. **g,** Proportion (mean±95% CI) of heritability (h_g^2) mediated by the *cis*-genetic component of five molecular phenotypes (h_{med}^2) in 34 tissues across GWAS traits. **h,** Heritability (estimated by MESC) of 16 complex traits of the pig with large sample sizes explained by independent molQTLs and those randomly MAF-matched SNPs. The top numerical labels are the *P* values based on the two-sided paired Student's *t*-test. **i,** Pearson's correlation (*r*) between snumber of colocalized molecular phenotypes and number of significant ($P < 1\times10^{-5}$) GWAS loci across traits. Each point represents each of the tested 268 GWAS traits. **j,** Pearson's correlation (*r*) between number of colocalized molecular phenotypes and tissue sample size. We only consider 14 tissues with a sample size of over 100. Each point represents a tissue. Error bar represents the 95% CI across all 268 tested GWAS traits. **k,** Pearson's correlation (*r*) between number of significant events (tissue-molecule pairs) from transcriptome-wide association studies (TWAS) by S-PrediXcan (single-tissue TWAS) and number of significant GWAS loci ($P < 1\times10^{-5}$) across 268 traits. Each point represents a tested GWAS trait. **l,** Pearson's correlation (*r*) between number of significant events (tissue-molecule pairs) from S-PrediXcan and those from S-MultiXcan (multi-tissue TWAS). **m,** Number of significant events (tissue-molecule pairs) in S-MultiXcan across 34 tissues. **n,** Pearson's correlation (*r*) between number of significant events (tissue-molecule pairs) by the summary-based Mendelian randomization (SMR) test and number of significant GWAS loci. **o,** Number of significant events (trait-molecule pairs, FDR < 0.05) in 268 traits by the SMR test across 34 tissues.

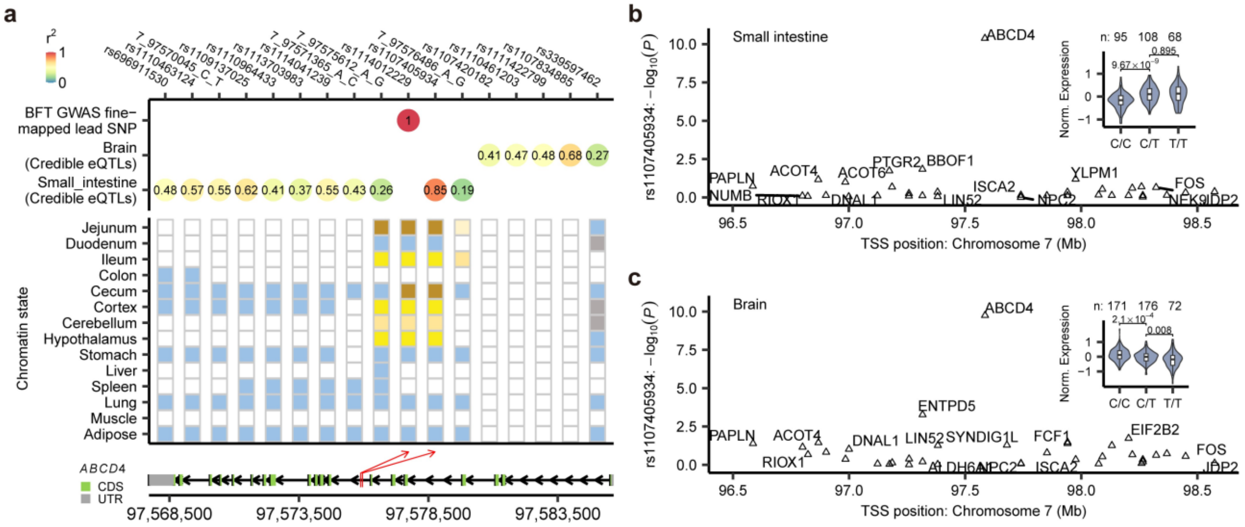

Supplementary Fig. 15 | Fine-mapping of the *ABCD4* locus for the BFT GWAS. a, The fine-mapped GWAS lead SNP (*rs1114012229*) is in high linkage disequilibrium (LD, r^2) with the credible eQTL (*rs1107405934*) in small intestine. SNPs *rs1114012229* and *rs1107405934* are located in enhancer regions in intestinal tissues and brain. **b,c,** Association *P*-values of *rs1107405934* with each gene within its *cis*-region in small intestine (**b**) and brain (**c**). The inner panels of (**b**) and (**c**) are normalized expression levels of *ABCD4* in the three genotypes of *rs1107405934*. The numerical labels between two violin plots are the *P* values that calculated from two-sided Student's *t*-test.

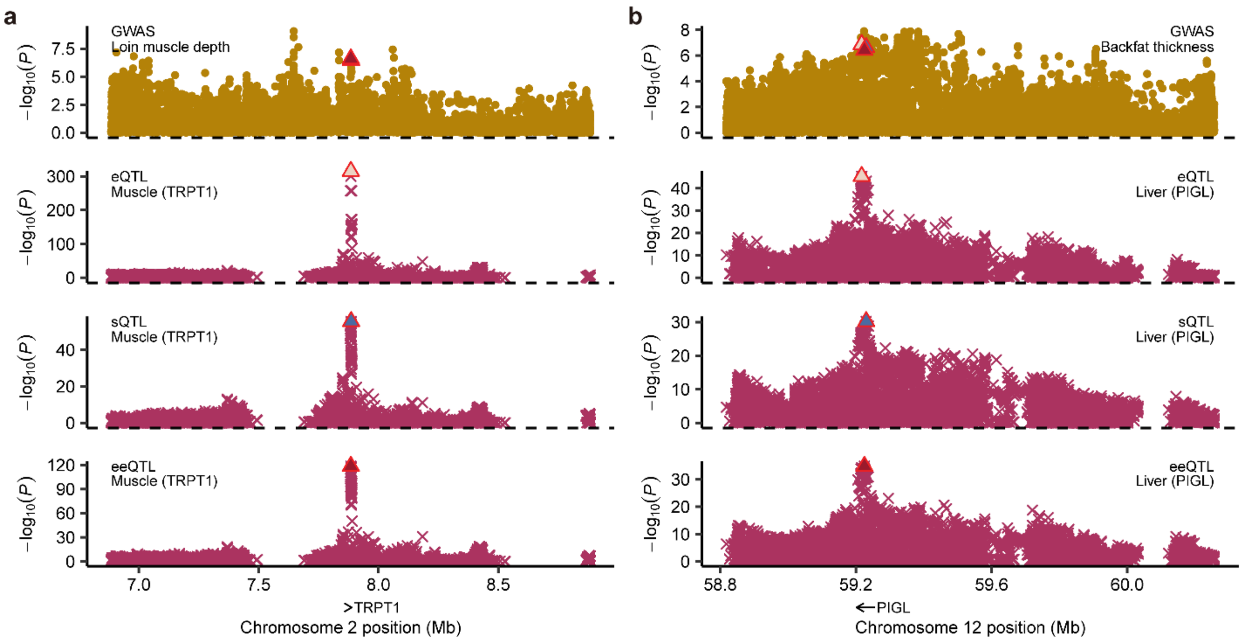

Supplementary Fig. 16 | Examples of GWAS loci colocalized with multiple types of molQTL. a, Significant SMR signals between GWAS loci of loin muscle depth and *cis*-eQTL, *cis*-sQTL, and *cis*-eeQTL of the *TRPT1* gene in muscle on chromosome 2. **b,** Significant SMR signals between GWAS loci of backfat thickness and *cis*-eQTL, *cis*-sQTL, and *cis*-eeQTL of the *PIGL* gene in liver on chromosome 12.

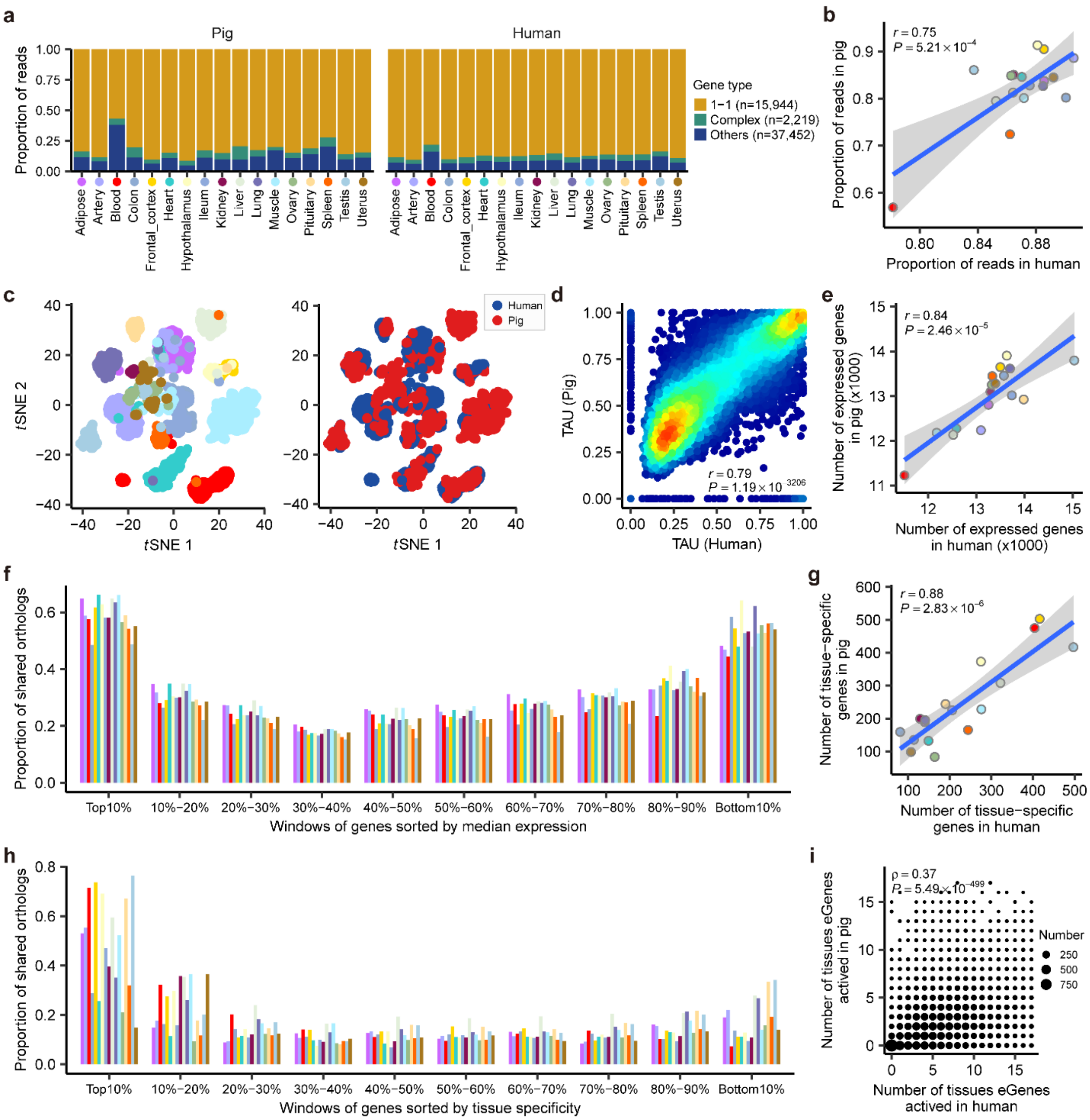

Supplementary Fig. 17 | Conservation of gene expression and eGenes between pigs and humans. a, Proportion of reads contributed by different types of genes regarding ortholog across 17 shared tissues in humans and pigs. 1-1 represents 1-to-1 orthologous genes between humans and pigs; Complex represents complex orthologous genes (1-many, many -1 and many-many); Others represents the rest of annotated genes in each species. **b,** Pearson's correlation (*r*) of proportion of reads contributed by 1-to-1 orthologous genes across 17 shared tissues between humans and pigs. Each dot represents a tissue with the same color key as (**a**). The line is fitted by a linear regression model using the *geom_smooth* function from ggplot2 (v3.3.2) in R (v4.0.2). The shading represents the standard error of the fitting line. **c,** *t*-SNE visualization of gene expression variation (based on 1-to-1 orthologous genes) among samples in humans (n=8,540) and pigs (n=3,913). Each dot represents one sample. The left panel is colored by tissue types

same as (**a**), while the right panel is colored by species. The gene expression data is integrated by Seurat v3.0. **d,** Pearson's correlation (*r*) of TAU values (measuring the tissue-specificity of gene expression) of 14,843 one-to-one orthologous genes between humans and pigs. Dots represent genes, and colors show the density of dots. **e,** Pearson's correlation (*r*) of number of expressed (Transcript per Million, TPM > 0.1) genes between humans and pigs. Each dot represents a tissue with the same color key as the *x*-axis of (**a**) panel. The line and shading are the same as in (**b**). **f,** Proportion of 1-to-1 orthologous genes shared between humans and pigs across 17 tissues. The genes are sorted by median expression from highest to lowest in each tissue each species. Each bar represents a tissue with the same color key as the *x*-axis of (**a**) panel. **g,** Pearson's correlation (*r*) of number of tissue-specific genes between humans and pigs across 17 tissues. Each dot represents a tissue with the same color key as the *x*-axis of (**a**) panel. The line and shading are the same as in (**b**). **h,** Proportion of 1-to-1 orthologous genes shared between humans and pigs across 17 tissues. The genes are sorted by tissue specificity (measured by *t*-statistics) from strongest to weakest in each tissue each species. **i,** Spearman's correlation (ρ) of number of tissues eGenes activated in between pigs and humans. Number is for number of eGenes.

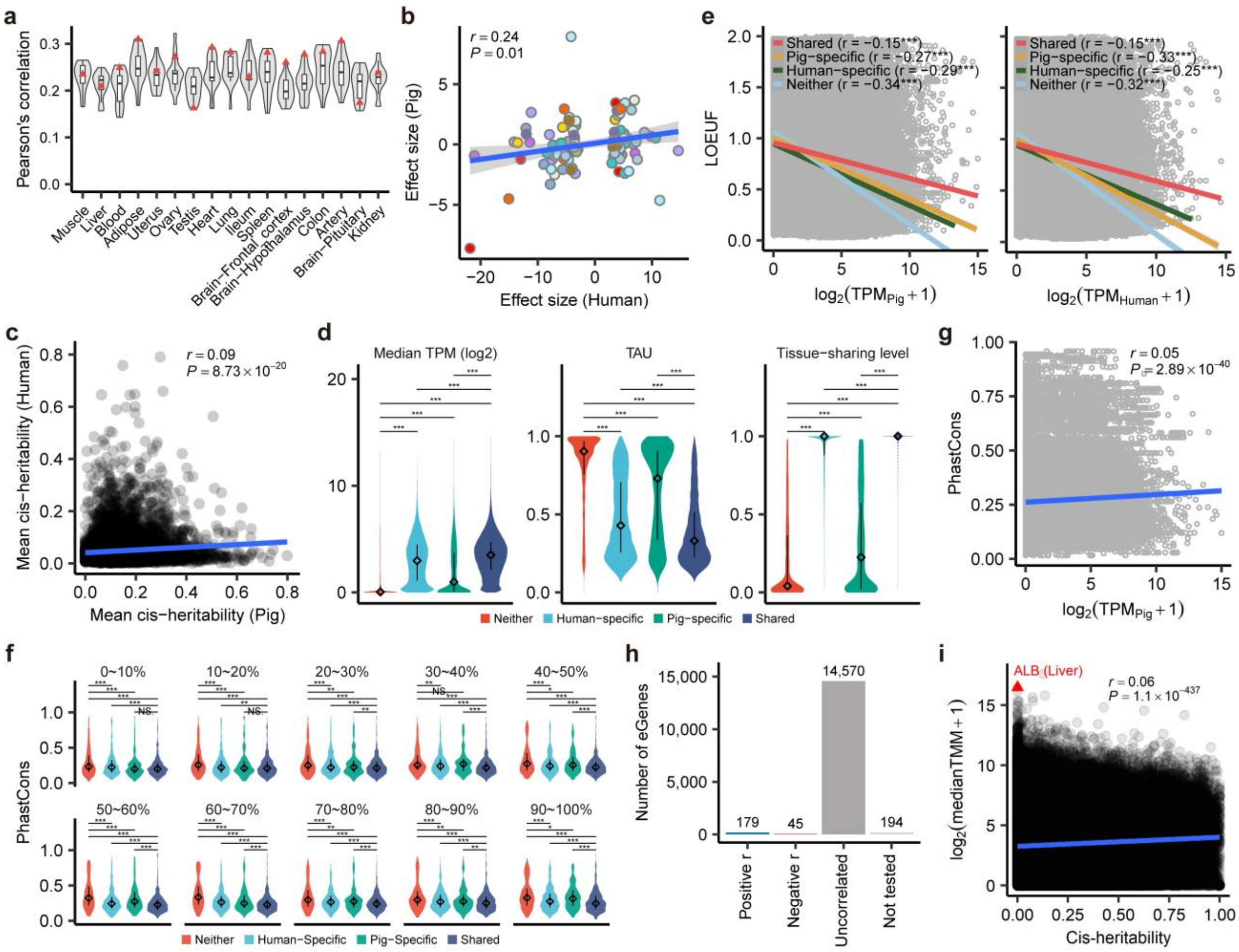

Supplementary Fig. 18 | Conservation of the regulator effects of genes and the genetic effects of complex traits between pigs and humans. a, Pearson's correlation of eQTL effect size in orthologous genes for each tissue. The red triangle represents the matching tissue between pigs and humans. **b,** Pearson's *r* of effects of 112 orthologous variants on gene expression between humans and pigs. Each point represents a variant, which is a significant *cis*-eQTL in humans. The line is fitted by a linear regression model using the *geom_smooth* function from ggplot2 (v3.3.2) in R (v4.0.2). The shading represents the standard error of the fitting line. **c,** Pearson's correlation of *cis*-heritability in orthologous genes between pig and human based on the average *cis*-heritability of each gene across human and pig in matching tissues. The line is the same as in (**b**). **d,** Expression levels (left), TAU values (middle) and tissue-sharing levels (right) for four groups of orthologous genes across 17 tissues in humans, including non-eGenes in neither species (Neither, n=3,993), human-specific eGenes (Human-specific, n=8,174), pig-specific eGenes (Pig-specific, n=3,882) and eGenes shared in both species (Shared, n=10,574). We defined the tissue-sharing level of an eGene as the proportion of the number of tissues that the eGene active in (LFSR < 0.05) across 49 human tissues. *** indicates the $P < 0.001$ that was obtained from the two-sided Wilcoxon rank-sum test. Diamond represents the median value and the error bar represents the upper and lower quartiles. **e,** Pearson's correlation between tolerance to loss of function mutations (LOEUF) and expression levels in pigs (left) and humans (right). The lines were fitted by the *geom_smooth* function in ggplot2. *** indicates $P < 0.001$ for the Pearson's correlation. **f,** PhastCons score in the four groups of orthologous genes in 10 evenly spaced expression level bins, where "0~10%"

represents the genes with the lowest 10% expression levels and “90~100%” represents the genes with top 10% expression levels. NS., *, ** and *** indicate one-sided Wilcoxon rank-sum test $P > 0.05$, $P < 0.05$, $P < 0.01$, and $P < 0.001$, respectively. **g,** Pearson’s correlation between PhastCons scores and expression levels of genes. The line is the same as in (**b**). **h,** Numbers of eGenes divided into four groups based on Pearson’s correlation between *cis*-eQTL effect size and eGene expression level across tissues. “Positive r” and “Negative r” represent genes with significant (FDR < 0.05) positive and negative correlations, respectively. “Uncorrelated” represents genes without significant correlations and “Not tested” represents those not tested owing to have not enough observations. **i,** Pearson’s correlation between *cis*-heritabilities and expression levels of genes. The *ALB* gene has a high expression level but its cis-heritability is zero in the liver. The line is the same as in (**b**).

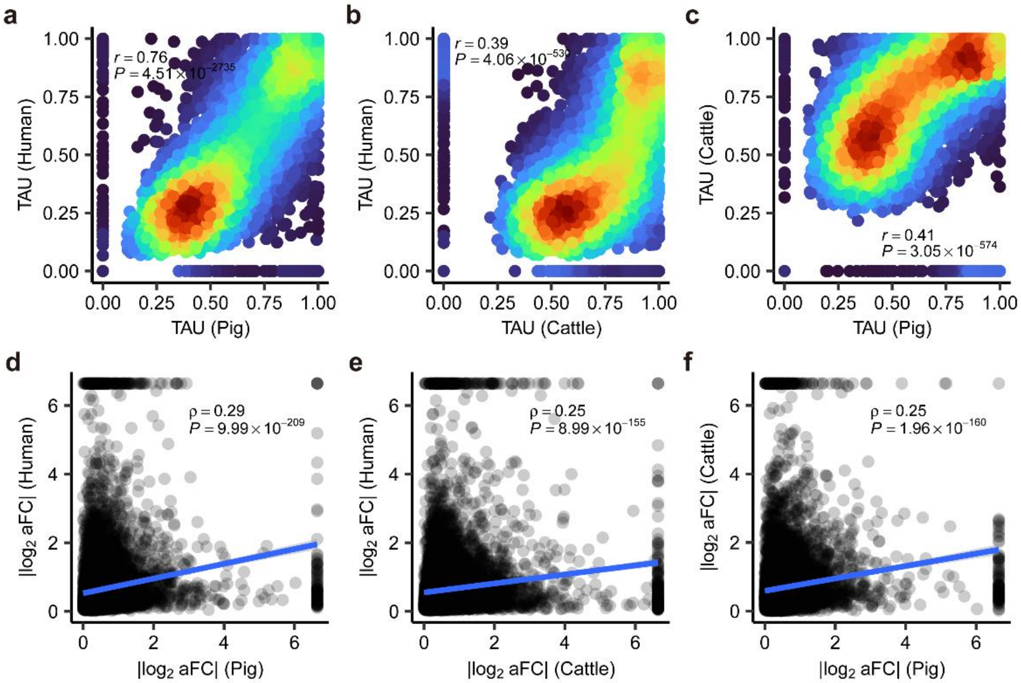

Supplementary Fig. 19 | Similarity of gene expression and eQTL genetic regulator effects across pigs, cattle, and humans. a-c, Pearson's correlation (*r*) of TAU values (measuring the tissue-specificity of gene expression) of 14,583 one-to-one orthologous genes between pigs, cattle, and humans. Dots represent genes, and colors show the density of dots. **d-f,** Spearman's correlation (ρ) of eQTL effect size $|\log_2(\mathrm{aFC})|$ in orthologous genes of 12 matched tissues between pigs, cattle, and humans. The lines are fitted by a linear regression model using the *geom_smooth* function from ggplot2 (v3.3.2) in R (v4.0.2).

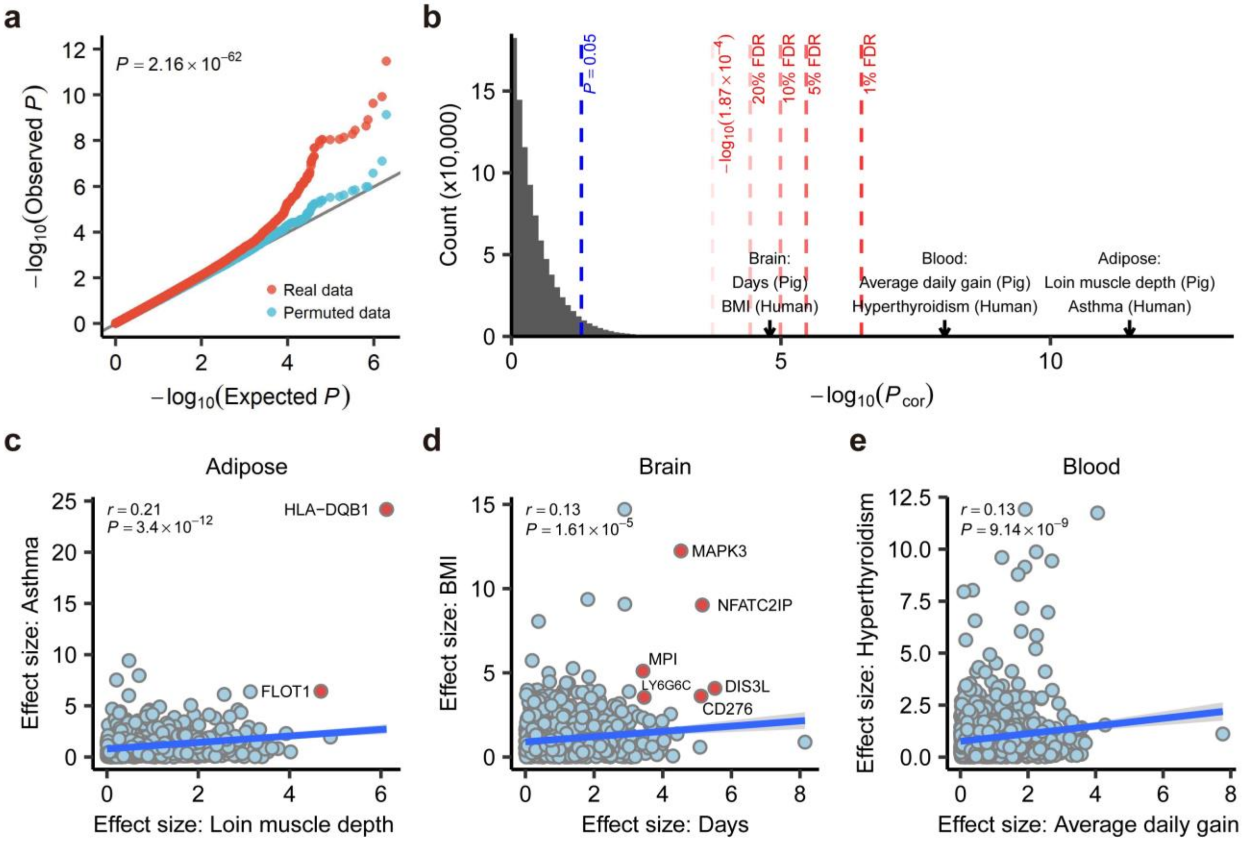

Supplementary Fig. 20 | Correlation of orthologous gene effect size between pig and human traits derived from transcriptome-wide association studies (TWAS). a, QQ-plot of *P*-values of TWAS correlations between pig and human (i.e., real data), compared to a permutation control (i.e., permuted data). *P* value were obtained by the two-sided Wilcoxon rank-sum test. **b,** Distribution of *P*-values from the TWAS correlations between pig and human. The blue line is the permutation corrected-$P = 0.05$, and the red lines are the *P*-values under different FDR cutoffs to adjust for multiple testing. **c,** Pearson's correlation (r) of effect sizes of 1,121 orthologous genes between loin muscle depth (pig) and asthma (human) traits in adipose. The *P* value is obtained from the Pearosn's correlation test. **d,** Effect sizes of 1,177 orthologous genes between days (pig) and BMI (human) in the brain. **e,** Effect sizes of 1,872 orthologous genes between average daily gain (pig) and hyperthyroidism (human) in blood. Dots represent orthologous genes. Red dots represent genes with significant (FDR < 0.05) TWAS signals in both species. The lines are fitted by a linear regression model. The lines are fitted by a linear regression model using the *geom_smooth* function from ggplot2 (v3.3.2) in R (v4.0.2). The shading represents the standard error of the fitting line.

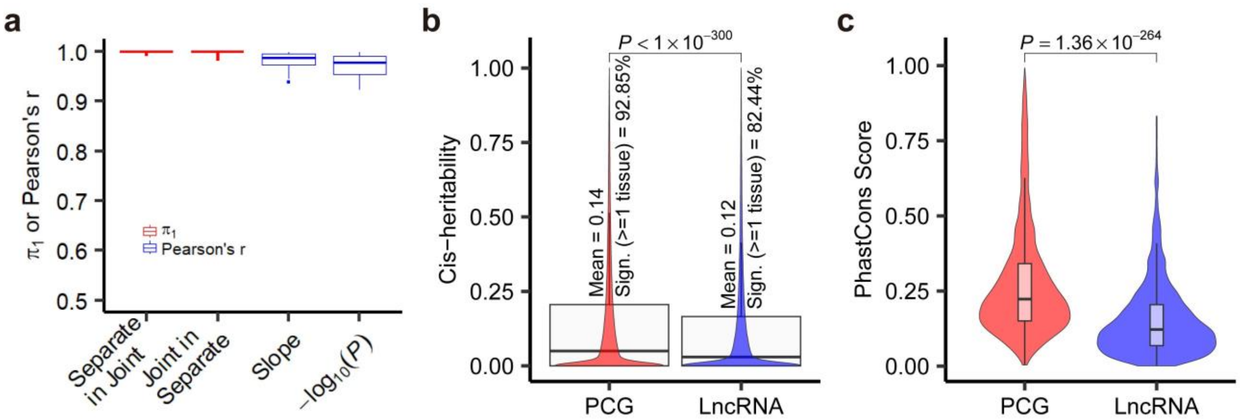

Supplementary Fig. 21 | Comparison of molQTLs discovery between separate and joint normalization for protein-coding genes (PCG) and lncRNAs. a, The replication rate (π_1) of eQTLs discovery between separate and joint normalization groups and the Pearson's correlation (*r*) of their summary statistics across 34 tissues. "Separate in Joint" represents the replication rate of eQTL of "Separate" in "Joint" and "Joint in Separate" represents the replication rate of eQTL of "Joint" in "Separate". "Separate" and "Joint" represent eQTL mapping using separate normalization and joint normalization for the expression of PCGs and lncRNAs, respectively. **b,c,** *Cis*-heritability (**b**) and PhastCons score (**c**) for PCGs and lncRNAs. *P*-values are obtained by two-sided Wilcoxon rank-sum tests.

Supplementary References

1. Krueger, F. & Andrews, S. R. Bismark: A flexible aligner and methylation caller for Bisulfite-Seq applications. *Bioinformatics* **27**, 1571–1572 (2011).

2. Liu, H. *et al.* Systematic identification and annotation of human methylation marks based on bisulfite sequencing methylomes reveals distinct roles of cell type-specific hypomethylation in the regulation of cell identity genes. *Nucleic Acids Res.* **44**, 75–94 (2016).

3. Song, Q. *et al.* A reference methylome database and analysis pipeline to facilitate integrative and comparative epigenomics. *PLoS One* **8**, e81148 (2013).

4. Camargo, A. P. Tspex : a Tissue-Specificity Calculator for Gene Expression Data. 1–7 (2020) doi:10.21203/rs.3.rs-51998/v1.

5. Ritchie, M. E. *et al.* Limma powers differential expression analyses for RNA-sequencing and microarray studies. *Nucleic Acids Res.* **43**, e47 (2015).

6. Pan, Z. *et al.* Pig genome functional annotation enhances the biological interpretation of complex traits and human disease. *Nat. Commun.* **12**, (2021).

7. Ernst, J. & Kellis, M. ChromHMM: Automating chromatin-state discovery and characterization. *Nat. Methods* **9**, 215–216 (2012).

8. Langfelder, P. & Horvath, S. WGCNA: An R package for weighted correlation network analysis. *BMC Bioinformatics* **9**, 559 (2008).

9. Hyvärinen, A. & Oja, E. Independent component analysis: Algorithms and applications. *Neural Networks* **13**, 411–430 (2000).

10. Stegle, O., Parts, L., Piipari, M., Winn, J. & Durbin, R. Using probabilistic estimation of expression residuals (PEER) to obtain increased power and interpretability of gene expression analyses. *Nat. Protoc.* **7**, 500–507 (2012).

11. Song, W. M. & Zhang, B. Multiscale Embedded Gene Co-expression Network Analysis. *PLoS Comput. Biol.* **11**, e1004574 (2015).

12. Russo, P. S. T. *et al.* CEMiTool: A Bioconductor package for performing comprehensive modular co-expression analyses. *BMC Bioinformatics* **19**, 56 (2018).

13. Kolberg, L., Kerimov, N., Peterson, H. & Alasoo, K. Co-expression analysis reveals interpretable gene modules controlled by trans-acting genetic variants. *Elife* **9**, 1–23 (2020).

14. Durinck, S., Spellman, P. T., Birney, E. & Huber, W. Mapping identifiers for the integration of genomic datasets with the R/ Bioconductor package biomaRt. *Nat. Protoc.* **4**, 1184–1191 (2009).

15. Wu, T. *et al.* clusterProfiler 4.0: A universal enrichment tool for interpreting omics data. *Innov.* **2**, 100141 (2021).

16. Leonard, M., Graham, S. & Bonacum, D. The human factor: The critical importance of effective teamwork and communication in providing safe care. *Qual. Saf. Heal. Care* **13**, 361–362 (2004).

17. Li, H. & Durbin, R. Fast and accurate short read alignment with Burrows-Wheeler transform. *Bioinformatics* **25**, 1754–1760 (2009).

18. Durand, N. C. *et al.* Juicer Provides a One-Click System for Analyzing Loop-Resolution Hi-C Experiments. *Cell Syst.* **3**, 95–98 (2016).

19. Lopez-Delisle, L. *et al.* pyGenomeTracks: reproducible plots for multivariate genomic datasets. *Bioinformatics* **37**, 422–423 (2021).

20. Wolff, J. *et al.* Galaxy HiCExplorer 3: A web server for reproducible Hi-C, capture Hi-C and single-cell Hi-C data analysis, quality control and visualization. *Nucleic Acids Res.* **48**, W177–W184 (2020).

21. Herrera-Uribe, J. *et al.* Reference Transcriptomes of Porcine Peripheral Immune Cells Created Through Bulk and Single-Cell RNA Sequencing. *Front. Genet.* **12**, 689406 (2021).

22. Butler, A., Hoffman, P., Smibert, P., Papalexi, E. & Satija, R. Integrating single-cell transcriptomic data across different conditions, technologies, and species. *Nat. Biotechnol.* **36**, 411–420 (2018).

23. Hao, Y. *et al.* Integrated analysis of multimodal single-cell data. *Cell* **184**, 3573-3587.e29 (2021).

24. Liu, C. *et al.* A portable and cost-effective microfluidic system for massively parallel single-cell transcriptome profiling. *bioRxiv* **4**, 818450 (2019).

25. Schiller, H. B. *et al.* The human lung cell atlas: A high-resolution reference map of the human lung in health and disease. *Am. J. Respir. Cell Mol. Biol.* **61**, 31–41 (2019).

26. Newman, A. M. *et al.* Determining cell type abundance and expression from bulk tissues with digital cytometry. *Nat. Biotechnol.* **37**, 773–782 (2019).

27. Bolger, A. M., Lohse, M. & Usadel, B. Trimmomatic: A flexible trimmer for Illumina sequence data. *Bioinformatics* **30**, 2114–2120 (2014).

28. Vinet, L. & Zhedanov, A. A 'missing' family of classical orthogonal polynomials. *J. Phys. A Math. Theor.* **44**, 1297–1303 (2011).

29. Li, H. A statistical framework for SNP calling, mutation discovery, association mapping and population genetical parameter estimation from sequencing data. *Bioinformatics* **27**, 2987–2993 (2011).

30. Browning, B. L., Zhou, Y. & Browning, S. R. A One-Penny Imputed Genome from Next-Generation Reference Panels. *Am. J. Hum. Genet.* **103**, 338–348 (2018).

31. Chang, C. C. *et al.* Second-generation PLINK: Rising to the challenge of larger and richer datasets. *Gigascience* **4**, 7 (2015).

32. Baes, C. F. *et al.* Evaluation of variant identification methods for whole genome sequencing data in dairy cattle. *BMC Genomics* **15**, 948 (2014).

33. Alexander, D. H., Novembre, J. & Lange, K. Fast model-based estimation of ancestry in unrelated individuals. *Genome Res.* **19**, 1655–1664 (2009).

34. Breiman, L. Random forests. *Mach. Learn.* **45**, 5–32 (2001).

35. de Goede, O. M. *et al.* Population-scale tissue transcriptomics maps long non-coding RNAs to complex disease. *Cell* **184**, 2633-2648.e19 (2021).

36. Yang, J., Lee, S. H., Goddard, M. E. & Visscher, P. M. GCTA: A tool for genome-wide complex trait analysis. *Am. J. Hum. Genet.* **88**, 76–82 (2011).

37. Mohammadi, P., Castel, S. E., Brown, A. A. & Lappalainen, T. Quantifying the regulatory effect size of cis-acting genetic variation using allelic fold change. *Genome Res.* **27**, 1872–1884 (2017).

38. Jiang, L. *et al.* A resource-efficient tool for mixed model association analysis of large-scale data. *Nat. Genet.* **51**, 1749–1755 (2019).

39. Ardlie, K. G. *et al.* The Genotype-Tissue Expression (GTEx) pilot analysis: Multitissue gene regulation in humans. *Science (80-.).* **348**, 648–660 (2015).

40. Storey, J. D. & Tibshirani, R. Statistical significance for genomewide studies. *Proc. Natl. Acad. Sci. U. S. A.* **100**, 9440–9445 (2003).

41. Crespo-Piazuelo, D. *et al.* Identification of transcriptional regulatory variants in pig duodenum, liver, and muscle tissues. *Gigascience* **12**, 1–14 (2022).

42. Urbut, S. M., Wang, G., Carbonetto, P. & Stephens, M. Flexible statistical methods for estimating and testing effects in genomic studies with multiple conditions. *Nat. Genet.* **51**, 187–195 (2019).

43. Aguet, F. *et al.* The GTEx Consortium atlas of genetic regulatory effects across human tissues. *Science (80-.).* **369**, 1318–1330 (2020).

44. Hu, Y. J., Sun, W., Tzeng, J. Y. & Perou, C. M. Proper Use of Allele-Specific Expression Improves Statistical Power for cis-eQTL Mapping with RNA-Seq Data. *J. Am. Stat. Assoc.* **110**, 962–974 (2015).

45. Vigorito, E. *et al.* Detection of quantitative trait loci from RNA-seq data with or without genotypes using BaseQTL. *Nat. Comput. Sci.* **1**, 421–432 (2021).

46. Castel, S. E., Mohammadi, P., Chung, W. K., Shen, Y. & Lappalainen, T. Rare variant phasing and haplotypic expression from RNA sequencing with phASER. *Nat. Commun.* **7**, (2016).

47. Pockrandt, C., Alzamel, M., Iliopoulos, C. S. & Reinert, K. GenMap: Ultra-fast computation of genome mappability. *Bioinformatics* **36**, 3687–3692 (2020).

48. Saha, A. & Battle, A. False positives in trans-eQTL and co-expression analyses arising from RNA-sequencing alignment errors. *F1000Research* **7**, 1–27 (2019).

49. Lee, B. T. *et al.* The UCSC Genome Browser database: 2022 update. *Nucleic Acids Res.* **50**, D1115–D1122 (2022).

50. Taylor-Weiner, A. *et al.* Scaling computational genomics to millions of individuals with GPUs. *Genome Biol.* **20**, 228 (2019).

51. Han, B. & Eskin, E. Interpreting meta-analyses of genome-wide association studies. *PLoS Genet.* **8**, (2012).

52. Davis, J. R. *et al.* An Efficient Multiple-Testing Adjustment for eQTL Studies that Accounts for Linkage Disequilibrium between Variants. *Am. J. Hum. Genet.* **98**, 216–224 (2016).

53. Castel, S. E., Levy-Moonshine, A., Mohammadi, P., Banks, E. & Lappalainen, T. Tools and best practices for data processing in allelic expression analysis. *Genome Biol.* **16**, 1–12 (2015).

54. Kim-Hellmuth, S. *et al.* Cell type–specific genetic regulation of gene expression across human tissues. *Science (80-.).* **369**, (2020).

55. Gabriel, S. B. *et al.* The structure of haplotype blocks in the human genome. *Science (80-.).* **296**, 2225–2229 (2002).

56. Neph, S. *et al.* BEDOPS: High-performance genomic feature operations. *Bioinformatics* **28**, 1919–1920 (2012).

57. Navarro Gonzalez, J. *et al.* The UCSC genome browser database: 2021 update. *Nucleic Acids Res.* **49**, D1046–D1057 (2021).

58. Mizuno, A. & Okada, Y. Biological characterization of expression quantitative trait loci (eQTLs) showing tissue-specific opposite directional effects. *Eur. J. Hum. Genet.* **27**, 1745–1756 (2019).

59. Liu, Y., Siegmund, K. D., Laird, P. W. & Berman, B. P. Bis-SNP: Combined DNA methylation and SNP calling for Bisulfite-seq data. *Genome Biol.* **13**, R61 (2012).

60. Ongen, H., Buil, A., Brown, A. A., Dermitzakis, E. T. & Delaneau, O. Fast and efficient QTL mapper for thousands of molecular phenotypes. *Bioinformatics* **32**, 1479–1485 (2016).

61. Cingolani, P. *et al.* A program for annotating and predicting the effects of single nucleotide polymorphisms, SnpEff: SNPs in the genome of Drosophila melanogaster strain w1118; iso-2; iso-3. *Fly (Austin).* **6**, 80–92 (2012).

62. Wen, X. Effective QTL Discovery Incorporating Genomic Annotations. *bioRxiv* 032003 (2015).

63. Cui, R. *et al.* Improving fine-mapping by modeling infinitesimal effects. *bioRxiv* 2022.10.21.513123 (2022).

64. Li, L. *et al.* An atlas of alternative polyadenylation quantitative trait loci contributing to complex trait and disease heritability. *Nat. Genet.* **53**, 994–1005 (2021).

65. Ramírez-Ayala, L. C. *et al.* Whole-genome sequencing reveals insights into the adaptation of French Charolais cattle to Cuban tropical conditions. *Genet. Sel. Evol.* **53**, 3 (2021).

66. Sandelin, A. JASPAR: an open-access database for eukaryotic transcription factor binding profiles. *Nucleic Acids Res.* **32**, 91D – 94 (2004).

67. Khamis, A. M. *et al.* A novel method for improved accuracy of transcription factor binding site prediction. *Nucleic Acids Res.* **46**, e72–e72 (2018).

68. KNÜPPEL, R., DIETZE, P., LEHNBERG, W., FRECH, K. & WINGENDER, E. TRANSFAC Retrieval Program: A Network Model Database of Eukaryotic Transcription Regulating Sequences and Proteins. *J. Comput. Biol.* **1**, 191–198 (1994).

69. Speed, D., Hemani, G., Johnson, M. R. & Balding, D. J. Improved heritability estimation from genome-wide SNPs. *Am. J. Hum. Genet.* **91**, 1011–1021 (2012).

70. Yao, D. W., O'Connor, L. J., Price, A. L. & Gusev, A. Quantifying genetic effects on disease mediated by assayed gene expression levels. *Nat. Genet.* **52**, 626–633 (2020).

71. Maaten, L.J.P. van der & Hinton, G.E. Visualizing High-Dimensional Data Using t-SNE. *J. Mach. Learn. Res.* **9**, 2579–2605 (2008).

72. Karczewski, K. J. *et al.* The mutational constraint spectrum quantified from variation in 141,456 humans. *Nature* **581**, 434–443 (2020).

73. Wheeler, H. E. *et al.* Survey of the Heritability and Sparse Architecture of Gene Expression Traits across Human Tissues. *PLOS Genet.* **12**, e1006423 (2016).
