# Supplementary material for: A compendium of genetic regulatory effects across pig tissues
Source: Nat Genet. 2024 Jan 4;56(1):112–23. doi: 10.1038/s41588-023-01585-7 (PMC10786720; doi:10.1038/s41588-023-01585-7)
Supplement: Supplementary file 2 — Reporting Summary [file 41588_2023_1585_MOESM2_ESM.pdf]

Corresponding author(s): Lingzhao Fang
Last updated by author(s): Oct 6, 2023

Reporting Summary

Nature Portfolio wishes to improve the reproducibility of the work that we publish. This form provides structure for consistency and transparency in reporting. For further information on Nature Portfolio policies, see our Editorial Policies and the Editorial Policy Checklist.

Statistics

For all statistical analyses, confirm that the following items are present in the figure legend, table legend, main text, or Methods section.

n/a	Confirmed	
☐	☒	The exact sample size (n) for each experimental group/condition, given as a discrete number and unit of measurement
☐	☒	A statement on whether measurements were taken from distinct samples or whether the same sample was measured repeatedly
☐	☒	The statistical test(s) used AND whether they are one- or two-sided<br>*Only common tests should be described solely by name; describe more complex techniques in the Methods section.*
☐	☒	A description of all covariates tested
☐	☒	A description of any assumptions or corrections, such as tests of normality and adjustment for multiple comparisons
☐	☒	A full description of the statistical parameters including central tendency (e.g. means) or other basic estimates (e.g. regression coefficient) AND variation (e.g. standard deviation) or associated estimates of uncertainty (e.g. confidence intervals)
☐	☒	For null hypothesis testing, the test statistic (e.g. F, t, r) with confidence intervals, effect sizes, degrees of freedom and P value noted<br>*Give P values as exact values whenever suitable.*
☐	☒	For Bayesian analysis, information on the choice of priors and Markov chain Monte Carlo settings
☐	☒	For hierarchical and complex designs, identification of the appropriate level for tests and full reporting of outcomes
☐	☒	Estimates of effect sizes (e.g. Cohen's d, Pearson's r), indicating how they were calculated

Our web collection on statistics for biologists contains articles on many of the points above.

Software and code

Policy information about availability of computer code

Data collection

All raw data analyzed in this study are publicly available for download without restrictions from SRA (https://www.ncbi.nlm.nih.gov/sra/) and BIGD (https://bigd.big.ac.cn/bioproject/) databases using the wget function in Linux. Details of RNA-Seq, WGS, WGBS, single-cell RNA-Seq and Hi-C datasets can be found in Supplementary Table 1, 2, 5, 8 and 9, respectively.

Data analysis

All the computational scripts and codes (with software version) for RNA-Seq, WGS, WGBS, single-cell RNA-Seq and Hi-C datasets analyses, as well as the respective quality control, molecular phenotype normalization, genotype imputation, molQTL mapping, functional enrichment, colocalization, SMR and TWAS are available at the FarmGTEx GitHub website (https://github.com/FarmGTEx/PigGTEx-Pipeline-v0, https://doi.org/10.6084/m9.figshare.24247771).

For RNA-Seq data analysis, we used Trimmomatic (v0.39), STAR (v2.7.0), Stringtie (v2.1.1), featureCounts (v1.5.2), Leafcutter (v0.2.9), GATK (v4.0.8.1), phASER (v1.1.1), and Beagle (v5.1) for quality control, mapping, gene expression quantification, alternative splicing, SNP calling, ASE analysis, and genotype imputation, respectively. For sample clustering, we used MEGA (vX) and then visualized with iTOL (v6). For tissue-specific gene expression, we used limma (v3.51.2). For gene co-expression analysis, we used WGCNA (v1.69), ICA (v1.0.2), PEER (v1.3), MEGENA (v1.3.7), and CEMiTool (v1.8.3). For gene functional enrichment analysis, we used clusterProfiler (v4.0) and visualized it using Gephi (v0.9.2).

For WGS analysis, we used Trimmomatic (v0.39), BWA-MEM (v0.7.5a-r405), Picard (v2.21.2), GATK (v4.1.4.1), and Beagle (v5.1) for quality control, mapping, marked duplicated reads, variants calling, and phasing, respectively. We used used PLINK (v1.90) to do LD pruning.

For WGBS, we used FastQC (v0.11.9), Trim Galore (v0.4.5), Bismark (v0.19.0), and SMART2 (v2.2.8), Methpipe (v4.1.1), and FastQTL (v2.184) for quality evaluation, quality control, read mapping and DNA methylation level extraction, hypomethylation region detection, allele-specific

methylation loci analysis and methylation QTL mapping, respectively.

For Hi-C, we used Trim Galore (v0.6.7), BWA (v0.7.17), Juicer (v1.6), Arrowhead (v1.22.01), hicConvertFormat (v3.7.1), pyGenomeTracks (v3.6) for quality control, read mapping, Hi-C contact matrix construction, TAD identification, format conversion, and visualization, respectively.

For single-cell RNA-Seq, we used Seurat (v3.0.2), Azimuth (v0.4.0) and CIBERSORTx online tool (v1) for data processing, cell type annotation and cell type deconvolution, respectively.

We removed SNPs with MAF < 0.01 and/or missing rate > 0.9 using bcftools (v1.9) and employed Beagle (v5.1) to phase the filtered variants and impute sporadically missing genotypes.

For QTL mapping, we used TensorQTL (v1.0.3), aFC (v0.3), dap-g (v1.0.0), METASOFT (v2.0.1), MashR (v0.2-6), and GCTA (v1.93.0) for cis-QTL mapping, effect size estimation, fine-mapping, meta-analysis, tissue-sharing pattern estimation, and cis-QTL mapping with mixed linear model, respectively. We estimated the genetic parameters using the restricted maximum likelihood (REML) method implemented in GCTA (v1.93.0).

We computed genotype PCs based on the filtered SNPs within each of the tissues using SNPRelate (v1.26.0). To account for technical confounders among RNA-Seq samples (e.g., hidden batch effects and other technical or biological factors), we used the Probabilistic Estimation of Expression Residuals (PEER) method, implemented in peer R package (v4.0.2), to estimate a set of latent covariates within each of the 34 tissues based on gene expression matrices. We computed the mappability of each locus in the reference genome using GenMap (v1.3.0). We removed SNPs in repeat regions annotated by the UCSC RepeatMasker track.

We first used imputed genotypes to estimate the ancestry composition of all RNA-Seq samples across tissues using ADMIXTURE (v1.3.0). we estimated the effect size (aFC) of the top ieQTL of ieGenes from ASE data using the script phaser_cis_var.py in phASER (v1.1.1)

For integrative analysis between GWAS and molQTL, we used S-PrediXcan and S-MultiXcan in MetaXcan (v0.6.11) for single-tissue and multi-tissue TWAS analysis, SMR (v1.03) for Mendelian Randomization analysis, and fastENLOC (v1.0) for colocalization. We performed a meta-analysis of molQTL across all 34 tissues using MashR (v0.2-6) and METASOFT (v2.0.1). We calculated the pairwise Rand index to measure the clustering similarity using the rand.index function in the fossil (v0.4.0) R package (v4.0.2).

we performed 2,056 separate GWAS, and conducted the meta-GWAS analysis for the same traits across different populations based on GWAS summary statistics using METAL (v2011-03-25).

For enrichment analysis, we used TORUS (v1) and ClusterProfiler (v4.0) for molQTLs and genes functional annotation, respectively.

For manuscripts utilizing custom algorithms or software that are central to the research but not yet described in published literature, software must be made available to editors and reviewers. We strongly encourage code deposition in a community repository (e.g. GitHub). See the Nature Portfolio guidelines for submitting code & software for further information.

Data

Policy information about availability of data

All manuscripts must include a data availability statement. This statement should provide the following information, where applicable:

- Accession codes, unique identifiers, or web links for publicly available datasets
- A description of any restrictions on data availability
- For clinical datasets or third party data, please ensure that the statement adheres to our policy

All raw data analyzed in this study are publicly available for download without restrictions from SRA (https://www.ncbi.nlm.nih.gov/sra/) and BIGD (https://bigd.big.ac.cn/bioproject/) databases. Details of RNA-Seq, WGS, WGBS, single-cell RNA-Seq and Hi-C datasets can be found in Supplementary Tables 1, 2, 5, 8 and 9, respectively. All WGS data generated in this study are available under CNCB GSA (https://ngdc.cncb.ac.cn/) accessions: PRJCA016120, PRJCA016130, PRJCA017284, PRJCA016012, and PRJCA016216. All processed data and the full summary statistics of molQTL mapping are available at http://piggtex.farmgtex.org/.

Human research participants

Policy information about studies involving human research participants and Sex and Gender in Research.

Reporting on sex and gender	NA
Population characteristics	NA
Recruitment	NA
Ethics oversight	NA

Note that full information on the approval of the study protocol must also be provided in the manuscript.

Field-specific reporting

Please select the one below that is the best fit for your research. If you are not sure, read the appropriate sections before making your selection.

☒ Life sciences ☐ Behavioural & social sciences ☐ Ecological, evolutionary & environmental sciences

For a reference copy of the document with all sections, see nature.com/documents/nr-reporting-summary-flat.pdf

Life sciences study design

All studies must disclose on these points even when the disclosure is negative.

Sample size

No power calculation was needed in advance in this study. In total, we analyzed all 11,323 RNA-Seq runs (downloaded by March, 2021) from SRA (https://www.ncbi.nlm.nih.gov/sra/), and BIGD databases (https://bigd.big.ac.cn/bioproject/), yielding 9,530 unique RNA-Seq samples. After filtering the samples with low quality (see below), all the remaining samples have been used for analysis.

Data exclusions

Full details of data exclusions for each analysis can be found in the Methods as well.

We filtered out RNA-Seq samples with clean read counts ≤ 500K or uniquely mapping rates < 60%, resulting in 8,262 samples. We further excluded samples with obvious clustering errors (e.g., samples labeled as liver that were not clustered with other liver samples), resulting in 7,095 samples for subsequent analysis.

For cis-QTLs detection, we excluded tissues with less than 40 individuals, resulting in 34 tissues for cis-QTL mapping.

Replication

To validate the cis-eQTLs, we applied four distinct strategies including linear mixed model, internal validation, external validation, and ASE validation.

First, we observed that the summary statistics of cis-eQTL derived from the linear regression model in TensorQTL had a strong correlation (an average Pearson's r of 0.91 across tissues) with those from a linear mixed model.

Second, we performed an internal validation in 18 tissues with over 80 samples by randomly dividing samples into two equal groups, and then conducting cis-eQTL mapping separately in both subgroups. We observed a high replication rate (an average $\pi1$ of 0.92) for cis-eQTL discovery.

Third, we found that 92%, 74%, 73%, and 69% of cis-eQTL in blood, liver, duodenum, and muscle, respectively, were replicated in independent datasets.

Fourth, we further found that effects (allelic fold changes, aFC) derived from allele specific expression (ASE) analysis were significantly correlated with those from cis-eQTL mapping consistently across tissues. For instance, in muscle, ASE-derived effects of 4,417 SNPs were significantly correlated (Spearman's $\rho = 0.76$, $P < 1e-300$) with their cis-eQTL effects.

Randomization

All the datasets are from observation studies and we used all samples publicly available after data exclusions listed above. Therefore, Randomization were not relevant in this study. Samples were grouped by tissue types.

Blinding

In this study, we re-analyzed all the publicly available RNA-seq data using a uniform pipeline, followed by the population-based association studies and validated the findings in independent populations. The blinding study design may be not applicable in this study.

Reporting for specific materials, systems and methods

We require information from authors about some types of materials, experimental systems and methods used in many studies. Here, indicate whether each material, system or method listed is relevant to your study. If you are not sure if a list item applies to your research, read the appropriate section before selecting a response.

Materials & experimental systems

n/a	Involved in the study	
☒	☐	Antibodies
☒	☐	Eukaryotic cell lines
☒	☐	Palaeontology and archaeology
☒	☐	Animals and other organisms
☒	☐	Clinical data
☒	☐	Dual use research of concern

Methods

n/a	Involved in the study	
☒	☐	ChIP-seq
☒	☐	Flow cytometry
☒	☐	MRI-based neuroimaging
